# Activation of STAT3 signaling is mediated by TFF1 silencing in gastric neoplasia

**DOI:** 10.1038/s41467-019-11011-4

**Published:** 2019-07-10

**Authors:** Mohammed Soutto, Zheng Chen, Ajaz A. Bhat, Lihong Wang, Shoumin Zhu, Ahmed Gomaa, Andreia Bates, Nadeem S. Bhat, Dunfa Peng, Abbes Belkhiri, M. Blanca Piazuelo, M. Kay Washington, Xi Chen Steven, Richard Peek, Wael El-Rifai

**Affiliations:** 1 Department of Veterans Affairs, Miami Healthcare System, Miami, FL USA; 2 Department of Surgery, University of Miami Miller School of Medicine, Miami, FL USA; 3 Division of Translational Medicine, Research Branch, Sidra Medicine, Doha, Qatar; 4 Department of Surgery, Vanderbilt University Medical Center, Nashville, TN USA; 5 Department of Medicine, Vanderbilt University Medical Center, Nashville, TN USA; 6 Department of Pathology, Vanderbilt University Medical Center, Nashville, TN USA; 7 Department of Public Health Sciences, Division of Biostatistics, University of Miami Miller School of Medicine, Miami, FL USA; 8 Sylvester Comprehensive Cancer Center, University of Miami Miller School of Medicine, Miami, FL USA

**Keywords:** Cancer, Cell biology, Gastroenterology

## Abstract

TFF1, a secreted protein, plays an essential role in keeping the integrity of gastric mucosa and its barrier function. Loss of TFF1 expression in the TFF1-knockout (KO) mouse leads to a pro-inflammatory phenotype with a cascade of gastric lesions that include low-grade dysplasia, high-grade dysplasia, and adenocarcinomas. In this study, we demonstrate nuclear localization of p-STAT^Y705^, with significant overexpression of several STAT3 target genes in gastric glands from the TFF1*-*KO mice. We also show frequent loss of TFF1 with nuclear localization of STAT3 in human gastric cancers. The reconstitution of TFF1 protein in human gastric cancer cells and 3D gastric glands organoids from TFF1-KO mice abrogates IL6-induced nuclear p-STAT3^Y705^ expression. Reconstitution of TFF1 inhibits IL6-induced STAT3 transcription activity, suppressing expression of its target genes. TFF1 blocks IL6Rα-GP130 complex formation through interfering with binding of IL6 to its receptor IL6Rα. These findings demonstrate a functional role of TFF1 in suppressing gastric tumorigenesis by impeding the IL6-STAT3 pro-inflammatory signaling axis.

## Abstract (web summary)

Trefoil factor 1 (TFF1) is a protein secreted by the gastric mucosa that protects against gastric tumourigenesis. Here, the authors show that TFF1 inhibits the oncogenic inflammatory response and IL-6-mediated STAT3 activation by interfering with the binding of IL6 to its receptor IL6Rα.

## Introduction

Gastric cancer remains one of the most common malignancies world-wide and the third leading cause of cancer-related deaths^1^. Interaction between genetic and environmental elements promote aberrant activation of oncogenic signaling pathways that drive cellular transformation and tumourigenesis^2,3^. Normal cellular homeostasis is achieved through a delicate coordinated balance between the activation and termination of signaling pathways, which subsequently control the timing and duration of a signal response. Loss or interruption of equilibrium of signaling activity leads to the development of abnormal cellular functions with acquisition of malignant properties, including increased cell proliferation, inflammation, and invasion^4^.

Signal Transducer and Activator of Transcription 3 (STAT3) belongs to a family of transcription factors that regulate expression of genes involved in both physiological and pathological cellular processes^5,6^. Constitutive activation of STAT3 protein is a common pro-inflammatory oncogenic feature identified in numerous solid tumors including gastric cancer^7,8^. STAT3 is directly phosphorylated and activated by the Janus kinase family of tyrosine kinases, which play a pivotal role in the development and progression of solid tumors^9^. In interleukin-6 (IL-6) signaling, the ligand binds its specific receptor, IL6Rα (also known as gp80), and generates heterodimeric IL-6–IL6Rα complexes that associate with two molecules of the signaling component of the IL-6 receptor, GP130, resulting in a hexameric structure that activates and phosphorylates glycoprotein 130 (GP130). Activation of GP130, also known as IL6 signal transducer (IL6ST), promotes the phosphorylation of Janus kinase 2 (JAK2), which in turn phosphorylates STAT3 on tyrosine-705. Phosphorylation of STAT3 results in STAT3 homodimerization, nuclear translocation, DNA binding, and transcription activation of STAT3 target genes^10,11^.

Trefoil factor 1 (TFF1), also known as pS2, is a small cysteine-rich acidic secreted protein of 60 amino acids expressed predominantly in the foveolar epithelial surface cells of the gastric mucosa^12^. TFF1 contains a trefoil domain that is defined as a sequence of 38 or 39 amino acid residues containing six cysteine residues which are disulfide-linked^13^. TFF1 plays a critical role in protecting the gastric mucosa integrity through formation of an intact mucus layer and mucosa barrier, as well as promoting mucosal repair after injury^14^. Loss of these protective mucosal functions in the TFF1-deficient mouse model promotes a multistep gastric tumourigenesis cascade with molecular features similar to human gastric cancer^15–17^. Silencing of TFF1 expression in the gastric mucosa can occur through a number of molecular mechanisms, including loss of heterozygosity (LOH), promoter methylation, and transcription suppression^18,19^. Recent studies suggest that TFF1 has anti-inflammatory and pro-apoptotic functions that protect the gastric mucosa and maintain its integrity, whereas its loss unlocks activation of oncogenic NF-κB and β-catenin signaling^16,20,21^.

In this study, we utilized human tissues, mouse models, organoid cultures and in vitro cell models to investigate the possible role of TFF1 in regulating STAT3 in gastric mucosa. The results demonstrate that TFF1 plays a critical role in suppressing IL6-mediated activation of oncogenic STAT3 through an interaction with IL6Rα, interfering with binding of IL6 to its receptor IL6Rα, and suppressing the formation of IL6Rα-GP130 complex.

## Results

### TFF1 loss promotes STAT3 activation in gastric tissues

**Fig. 1:**
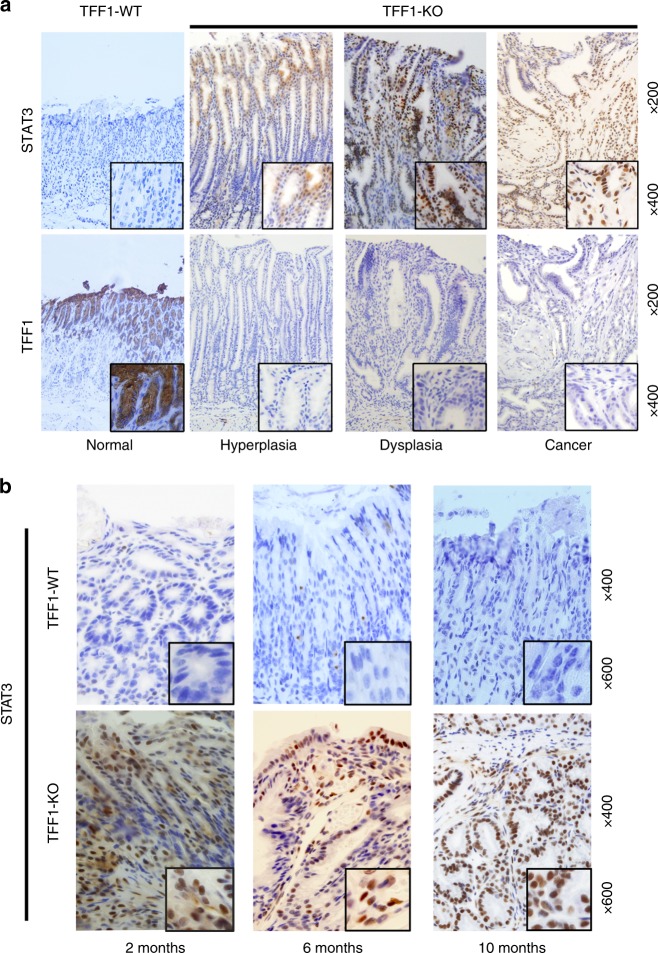
Loss of TFF1 promotes STAT3 nuclear localization in the TFF1-KO mice gastric tissues. **a** Representative immunohistochemistry images in the upper panels show nuclear p-STAT3 (Y705) in hyperplasia, dysplasia, and cancer from the antropyloric region of glandular stomach in TFF1-knockout (TFF1*-*KO), but not in TFF1*-*wild-type (TFF1*-*WT) normal gastric mucosa. The lower panels demonstrate the expression of TFF1, original magnification (×200) and insets (×400). **b** Immunohistochemistry staining of p-STAT3 of gastric mouse tissues from TFF1-WT and TFF1-KO mice at the age of 2, 6, and 10 months detected p-STAT3 in the nucleus at all ages in TFF1-KO mice. p-STAT3 staining was absent in tissues from matched ages in TFF1-WT mice with normal glands. The TFF1-KO mice showed dysplastic lesions at 6 and 10 months of age. Original magnification (×400) and insets (×600)

Our recent molecular analysis study using integrative bioinformatics suggested activation of STAT3 transcription network as a prominent feature of gastric cancer in mouse and human tissues^17^. Using Gene Set Enrichment Analysis (GSEA) to analyze gene-expression profiles^17^, we found that STAT3-associated genes were significantly enriched in the TFF1-KO mouse (FDR *=* 0.00749*, P* = 0.00069) and human gastric cancer samples (FDR = 0.00174; *P* = 0.00022) (Supplementary Fig. 1). Using phospho-STAT3 (Y705) antibody, immunohistochemistry (IHC) staining analysis demonstrated nuclear immunostaining of phospho-STAT3 in nondysplastic, dysplastic, and invasive adenocarcinoma lesions at the antropyloric region of the stomach in the TFF1-KO mice (Fig. 1a, upper panels). In contrast, nuclear staining for phospho-STAT3 was not detected in the same region of the stomach in the TFF1-WT mice (Fig. 1a, upper panel). As expected, TFF1 staining was positive in TFF1-WT and negative in TFF1-KO mouse gastric tissues (Fig. 1a, lower panels). Next, we evaluated the levels of nuclear STAT3 across a range of ages from 2, 6, and 10 months. The results indicated that nuclear accumulation of STAT3 in the stomach was present in TFF1-KO mice as early as 2 months of age, before the onset of dysplastic lesions (Fig. 1b and Supplementary Fig. 2a). The stomach tissues from TFF1-WT mice showed normal histology and lacked nuclear staining of p-STAT3 at all ages (Fig. 1a and Supplementary Fig. 2a, b). IHC data were confirmed by Western blot analysis, using cell lysate from mouse gastric tissues. The results demonstrated phosphorylation of STAT3 in TFF1-KO and its absence in TFF1-WT (Supplementary Fig. 2c). These results indicate that nuclear localization of STAT3 is present prior to the onset of dysplastic lesions in the TFF1*-*KO mice, suggesting a causal relationship between loss of TFF1 and activation of STAT3.

### TFF1 loss upregulates STAT3 target genes in gastric tissues

**Fig. 2:**
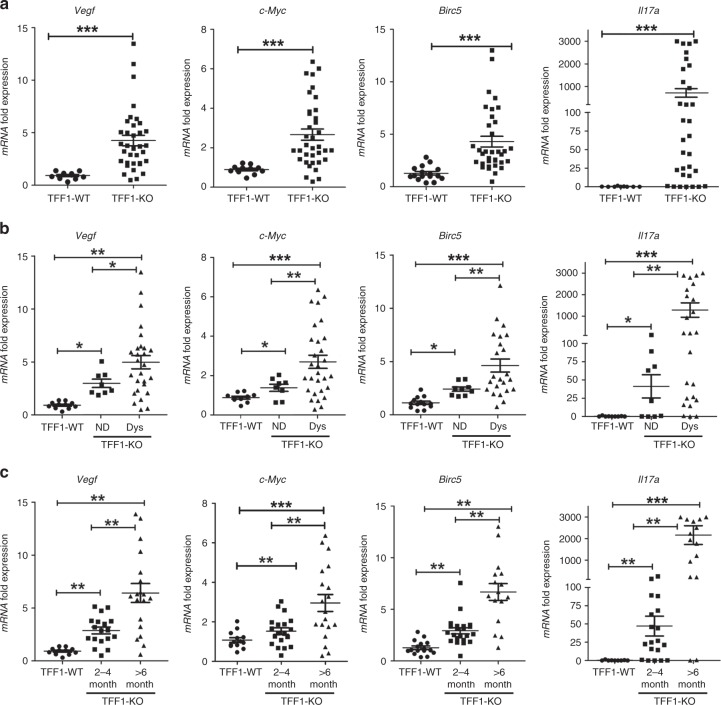
Loss of TFF1 promotes mRNA expression of STAT3 target genes in gastric tumourigenesis. **a** Quantitative real-time PCR analysis demonstrated upregulation of mRNA expression of STAT3 target genes (*Vegf*, *c-Myc*, *Birc5*, and *Il17A*) in gastric tissues from the TFF1-KO mice (*n* = 36) as compared with normal gastric tissues from TFF1-WT mice (*n* = 15). **b** Expression levels of STAT3 target genes in nondysplastic and dysplastic gastric mucosa from TFF1-KO, as compared to normal gastric mucosa from TFF1*-*WT. **c** Expression levels of STAT3 target genes in TFF1-KO gastric mucosa at 2–4 and >6 months of age, as compared to age-matched *TFF1-*WT gastric mucosa. Horizontal bars indicate the mean values. ^*^P < 0.05, ^**^P < 0.01 and ^***^P < 0.001 by two-tailed Student’s *t* test (for two groups) and ANOVA Newman–Keuls multiple comparison test (for multiple groups)

To confirm transcription activation of nuclear STAT3 in vivo, we analyzed the mRNA expression levels of several known STAT3 targets genes, using tissues from the stomach of TFF1-KO and TFF1-WT mice (oligonucleotide sequences are provided in Supplementary Table 1). The quantitative real-time polymerase chain reaction (RT-PCR) (quantitative RT-PCR (qRT-PCR)) results showed a significant increase of mRNA expression levels of *Vegf* (*P* < 0.001), *c-Myc (P* < 0.05)*, Birc5 (P* < 0.001), and *Il17a* (*P* < 0.001) in TFF1-KO mouse gastric tissues, as compared to TFF1-WT (Fig. 2a). Our data indicated that overexpression of STAT3 target genes occurred in nondysplastic tissues (*P* < 0.05), with additional increase in gastric dysplasia (*P* < 0.01) (Fig. 2b). A significant and progressive increase in the expression of STAT3 target genes was observed with aging in the TFF1-KO mice, as compared to TFF1-WT (*P* < 0.01) (Fig. 2c); consistent with the expected progressive changes in histological lesions. Gastric tissues from the TFF1-WT mice did not display any significant changes in expression of STAT3 target genes at different ages (Supplementary Fig. 3). We also analyzed additional STAT3 target genes (*Il23*, *Il11*, *Il6*, *Ccl2*, *Ccl3*, and *Bcl2*) and revealed similar findings showing their increased expression in TFF1-KO as compared to TFF1-WT (Supplementary Fig. 4e, f, *P* < 0.01). Collectively, the qRT-PCR data, together with IHC and immunofluorescence results, suggest that loss of TFF1 promotes phosphorylation, nuclear localization, and transcription activation of STAT3 in the glandular stomach of the TFF1-KO mouse model.

### TFF1 regulates STAT3 activity in ex vivo mouse gastric tissues

**Fig. 3:**
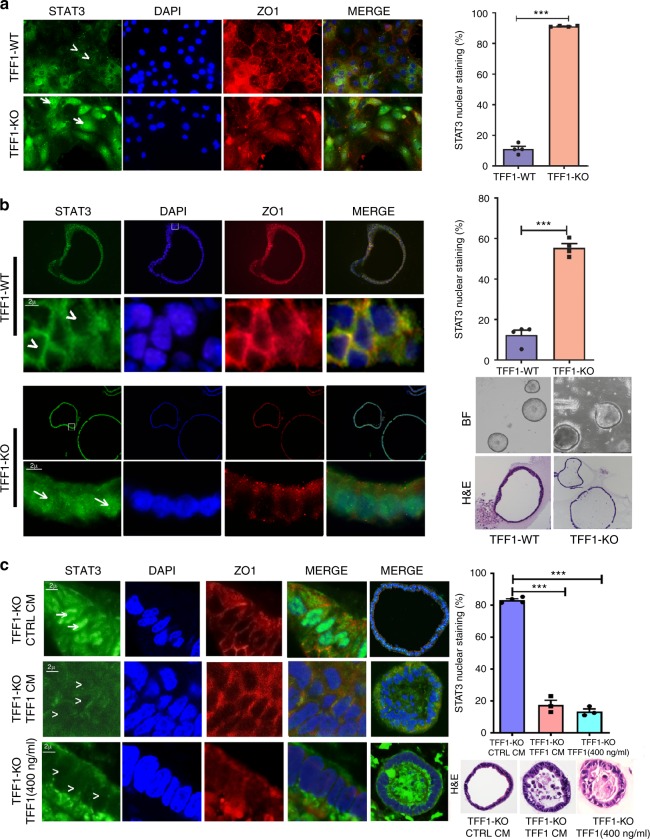
TFF1 suppresses STAT3 nuclear localization in ex vivo and 3D organoid cultures. **a** Ex vivo immunofluorescence staining of p-STAT3 (Y 705) in gastric epithelial cells isolated from the antropyloric region in TFF1-wild type (TFF1-WT) or TFF1-knockout (TFF1-KO) mice. As shown, there was absence of nuclear p-STAT3 staining in TFF1-WT cells (arrowheads in upper panels), whereas strong nuclear p-STAT3 was detected in TFF1-KO (arrows in lower panels). Quantification of nuclear p-STAT3 positive staining in at least 200 counted cells from three different fields is presented as percentage ± SEM (right panel); ^***^P < 0.001 by two-tailed Student’s *t* test. **b** 3D organoid cultures derived from antropyloric glands of TFF1-WT (upper two panels) and TFF1-KO (lower two panels) showing nuclear localization of p-STAT3 in TFF1-KO organoids (arrows), but not in TFF1-WT (arrowheads). **c** Immunofluorescence assay performed on organoids derived from the antrum of TFF1-KO mouse stomach. Cells showing nuclear staining of p-STAT3 in organoids treated with conditioned media from AGS-pcDNA cell line (Ctrl CM) for 24 h (upper panel). Organoids treated with TFF1 conditioned media from AGS-TFF1 cell line (TFF1 CM) displayed loss of nuclear staining (arrowheads) of p-STAT3 (middle panel). Organoids treated with recombinant TFF1 protein (400 ng mL^−1^) showed absence of p-STAT3 (arrowheads) nuclear staining (lower panels). The merge of the representative full organoid gland and its H&E staining is presented in the right panels. Scale bar 2 µm. Original magnification is ×600. Zo1 (red) was used as an epithelial cell marker, and DAPI (blue), as a nuclear counterstain. Graph showing the quantification of nuclear p-STAT3-positive cells in at least 4 counted organoid glands (at least 25–50 cells in each organoid) presented as percentage ± SEM (right upper panel); ^***^P < 0.001 by two-tailed Student’s *t* test. H&E staining and bright field images (BF) of representative organoids are shown on the right of panels (**b**) and (**c**)

Using ex vivo cultures of primary gastric epithelial cells and 3D organoid cultures derived from the antropyloric region of TFF1-WT and TFF1-KO mouse gastric tissues, we evaluated the baseline expression and nuclear localization of STAT3. The ex vivo immunofluorescence analysis demonstrated a significant increase of nuclear staining of STAT3 in primary gastric epithelial cells (Fig. 3a), and in 3D culture of organoids derived from TFF1-KO gastric mouse tissue, as compared to TFF1-WT (Fig. 3b, *P* < 0.001).

To determine if TFF1 loss mediates activation of STAT3, we treated organoids derived from TFF1-KO mice with conditioned media from AGS gastric cancer cell lines expressing TFF1 or recombinant TFF1 (400 ng mL^−1^) for 24 h, and compared them to TFF1-KO organoids treated with condition media from AGS cells expressing empty vector (control). Immunofluorescence analysis indicated that treatment with AGS-TFF1-conditioned media or TFF1 recombinant protein abrogated STAT3 nuclear staining (Fig. 3c, *P* < 0.001). Taken together, these data suggest that the secreted form of TFF1 inhibits nuclear localization and activation of STAT3 in gastric cells.

### TFF1 suppresses IL6-mediated STAT3 activation

**Fig. 4:**
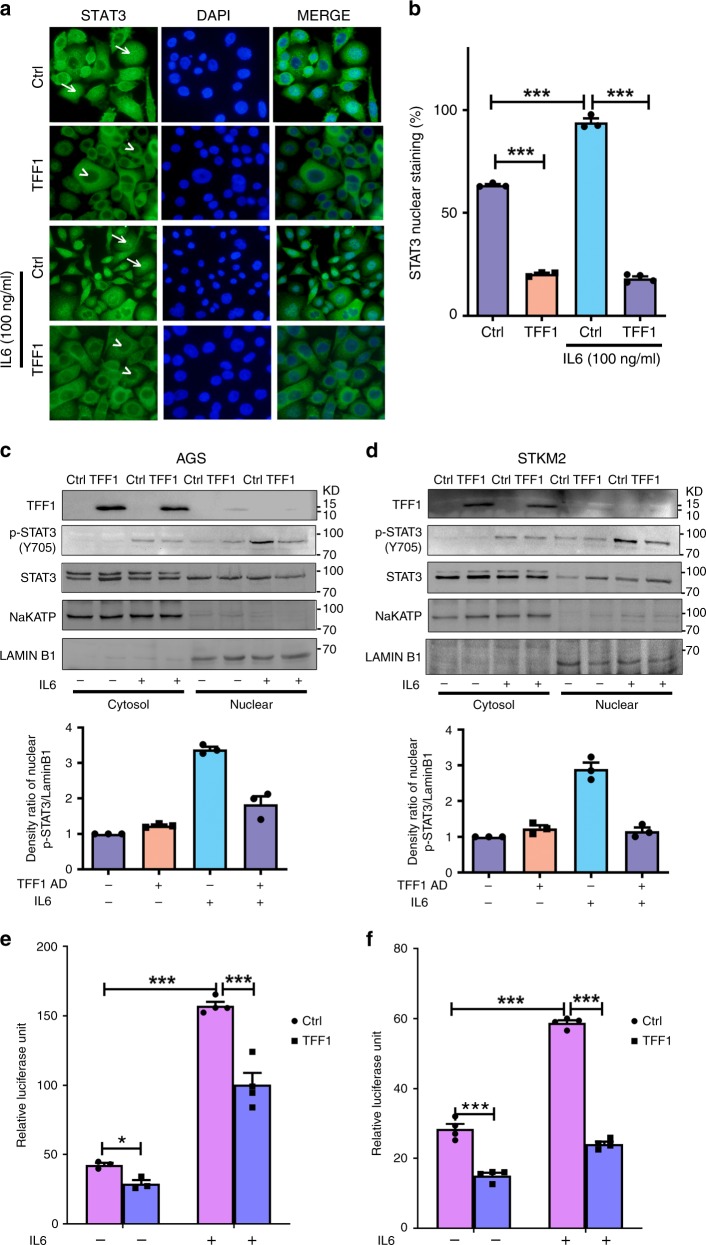
Reconstitution of TFF1 inhibits IL6-mediated STAT3 activation. **a** In vitro immunofluorescence assay of AGS pcDNA control cells (Ctrl) and AGS cells stably expressing pcDNA-TFF1. Cells were cultured and treated with or without IL6 (100 ng mL^−1^). Nuclear localization of STAT3 is shown in green (arrows). DAPI (blue) was used as a nuclear counterstain. Original magnification is at ×400. **b** Quantification of nuclear STAT3-positive staining in at least 200 cells from three images is presented as a percentage in the right panel. Data are graphed with mean ± SEM. **c**, **d** Protein-expression levels from the nuclear and cytosolic fractions of AGS (**c**) and STKM2 (**d**) cells infected with control or TFF1 adenoviruses (5 MOI). After 48 h, cells were treated or not with IL6 (100 ng mL^−1^) for 30 min. Similar amounts of protein were applied to SDS-PAGE for immunoblotting with total and p-STAT3 (Y705) antibodies. Anti-NaKATPase antibody was used as a loading control for cytosol fraction, and anti-LAMIN B1 for nuclear fraction. The relative intensity ratio of nuclear p-STAT3/Lamin B1 is calculated and graphed using Image-lab software from BioRad (lower panels). **e**, **f** The luciferase reporter assay using a STAT3–Luc reporter plasmid. The results are expressed as mean ± SEM of at least three independent experiments. AGS cells (**e**) and STKM2 cells (**f**) infected with control or TFF1 adenoviruses were transfected with STAT3–luciferase reporter and stimulated with IL6 (100 ng mL^−1^) for 3 h. The results are expressed as mean ± SEM of at least three independent experiments; ^*^P < 0.05, ^**^P < 0.01, and ^***^P < 0.001 by ANOVA Newman–Keuls multiple comparison test

Because of the known role of IL6 cytokine in the activation of STAT3 pathway, we next examined if TFF1 can interfere with IL6-mediated activation of STAT3. Our data indicated that stable reconstitution of TFF1 in AGS cells can significantly decrease the expression of nuclear STAT3, as compared to control cells. Stimulation of AGS cells with IL6 (100 ng mL^−1^) for 6 h led to a significant increase in nuclear localization of STAT3, whereas stable reconstitution of TFF1 significantly suppressed IL6-mediated nuclear localization of STAT3 (Fig. 4a, b, *P* < 0.001). We also confirmed these findings using tissue organoids derived from TFF1-WT and TFF1-KO gastric mucosa, following treatment with IL6 (100 ng mL^−1^) for 6 h. Immunofluorescence data indicated that IL6 treatment could not induce nuclear localization of STAT3 in TFF1-WT 3D organoid cultures (Supplementary Fig. 5a, b–e); TFF1-KO organoids were used as a control for STAT3 activation. These data clearly indicates that TFF1 suppresses IL6-mediated STAT3 nuclear localization.

To confirm the immunofluorescence data, we examined the levels of STAT3 phosphorylation in cytosol and nuclear fractions, using Western blot analysis. AGS and STKM2 cells were infected with 5 MOI (multiplicity of Infection) of TFF1 adenovirus or control adenovirus for 48 h and stimulated or not with IL6 for 30 min; 5 MOI of TFF1 was identified as the optimum dose, as shown in Supplementary Fig. 6a. The results indicated that TFF1 reconstitution suppressed IL6-mediated nuclear phosphorylation of STAT3 (Y705) in both cell lines (Fig. 4c, d, *P* < 0.001)*.*

Given the fact that TFF1 is a secreted protein, AGS gastric cancer cell lines were treated with TFF1 recombinant protein (400 ng mL^−1^) for 24 h, followed by stimulation with IL6 (100 ng mL^−1^) for 30 min. The results demonstrated a notable increase of phospho-STAT3 (Y705) in untreated AGS cells, an effect that was reduced after adding recombinant TFF1 (Supplementary Fig. 7a); the optimal dose effect of recombinant TFF1 on phospho-STAT3 was determined in AGS cells (Supplementary Fig. 6b). Similar results were obtained using a transient tet-inducible TFF1 expression system (AGS-TFF1 TetOne stable cells) (Supplementary Fig. 7b).

Next, we evaluated the effect of TFF1 on the transcriptional activity of STAT3 using luciferase reporter assay. AGS and STKM2 cells were transiently infected with control adenovirus or TFF1 adenovirus particles (five MOI). Next day, the cells were transfected with STAT3-Luc. After 24 h incubation, the cells were stimulated with IL6 (100 ng mL^−1^) for 3 h and STAT3-Luc activity was measured. The data indicated that STAT3-Luc activity was significantly reduced in cells expressing TFF1 adenovirus, as compared to control adenovirus (Fig. 4e, f, *P* < 0.001). Similar results were obtained using TFF1 recombinant protein and AGS-TetOne cells expressing TFF1 (Supplementary Fig. 7c, d, *P* < 0.001).

To confirm the specific role of TFF1 in decreasing activation and phosphorylation of STAT3, AGS cells were treated with TFF1 recombinant protein (400 ng mL^−1^), with or without overnight incubation with neutralizing antibody against TFF1. The following day, cells were treated with IL6 for 30 min and proteins were collected for Western blot analysis. The results, as expected, confirmed the decrease of phospho-STAT3 following IL6 stimulation in cells treated with recombinant TFF1 protein, as compared to control cells. On the other hand, incubation of cells with TFF1 neutralizing antibody together with TFF1 recombinant protein, abrogated the inhibitory effect of TFF1 on phospho-STAT3 (Supplementary Fig. 8). These results clearly indicate the specificity of TFF1 in suppressing activation and phosphorylation of STAT3 in gastric cancer cells. Collectively, by immunofluorescence, Western blot analysis and STAT3–luc reporter assays, our data indicates TFF1 regulates the phosphorylation and transcription activation of STAT3.

### TFF1 suppresses activation of STAT3 target genes in vitro

**Fig. 5:**
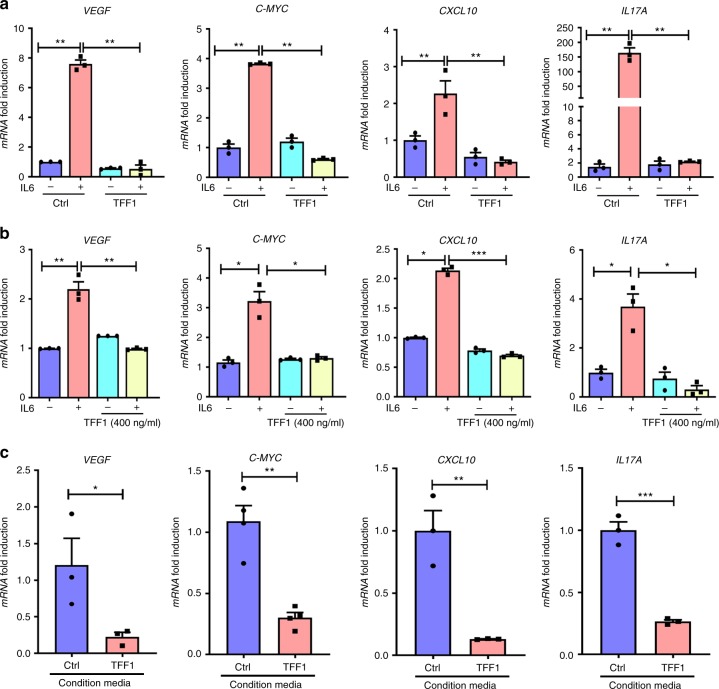
TFF1 suppresses expression of STAT3 target genes in vitro. **a**–**c** Quantitative real-time PCR analysis demonstrated expression levels of several STAT3 target genes (*VEGF*, *C-MYC*, *CXCL10*, and *IL17A*) in vitro. **a** AGS cell lines were infected with either TFF1 or control adenoviruses (5 MOI) for 48 h and then treated with or without IL6 (100 ng mL^−1^) for 2 h. **b** AGS cells were treated with or without TFF1 recombinant protein (400 ng mL^−1^) for 24 h and either stimulated or not with IL6 for 2 h. **c** AGS cells were treated with conditioned media from AGS-pcDNA or AGS-TFF1 stable cell lines for 24 h. The results are expressed as mean ± SEM of at least 3 independent experiments; ^*^P < 0.05, ^**^P < 0.01 and ^***^P < 0.001 by two-tailed Student’s *t* test (for two groups) and ANOVA Newman–Keuls multiple comparison test (for multiple groups)

Using qRT-PCR, we determined the mRNA expression level of several STAT3 target genes (*VEGF*, *C-MYC*, *CXCL10*, and *IL17A*) following TFF1 reconstitution and stimulation with IL6 (oligonucleotide sequences are provided in Supplementary Table 1). AGS cells were infected with control adenovirus or TFF1 adenovirus (5 MOI) for 48 h followed by stimulation with IL6 (100 ng mL^−1^) for 2 h. The qRT-PCR results revealed a significant increase of mRNA expression of the aforementioned STAT3 target genes, following IL6 stimulation in AGS control cells (Fig. 5a). This increase was significantly abolished with the reconstitution of TFF1 (Fig. 5a). Similar results were obtained using TFF1 recombinant protein (400 ng mL^−1^) for 24 h, followed by 2 h treatment with IL6 (Fig. 5b). We also used TFF1 conditioned media, collected from AGS cells stably expressing TFF1 or control empty vector, and determined the expression of STAT3 target genes. Our findings revealed reduced expression of these targets in cells treated with TFF1 conditioned media, as compared to control media (Fig. 5c).

**Fig. 6:**
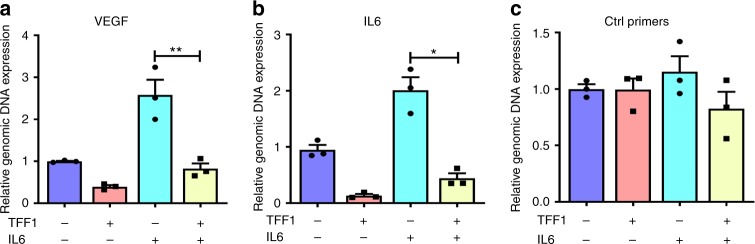
ChIP assay confirms the decreased binding of STAT3 to its target genes after TFF1 reconstitution. **a**–**c** ChIP assay in AGS cells infected with control or TFF1 adenoviruses and stimulated with or without IL6 (100 ng mL^−1^), followed by quantitative real-time PCR with primers designed for STAT3 binding site on *VEGF* (**a**) and *IL6* (**b**) promoter regions and control primers (**c**)

To confirm the decreased binding of STAT3 to its target genes after TFF1 expression, we performed quantitative chromatin immunoprecipitation (ChIP) assay for two known STAT3 target genes, *VEGF* and *IL6*. By using qRT-PCR, we detected a significant decrease of STAT3 recruitment to the VEGF (Fig. 6a, *P* < 0.01) and *IL6* (Fig. 6b *P* < 0.05) promoters in AGS-TFF1 expressing cells, upon stimulation with IL6. Altogether, these data indicate that TFF1 can suppress IL6-mediated STAT3 activity, binding, and transcription of its target genes.

### TFF1 inhibits IL6–IL6Rα–GP130 complex formation

**Fig. 7:**
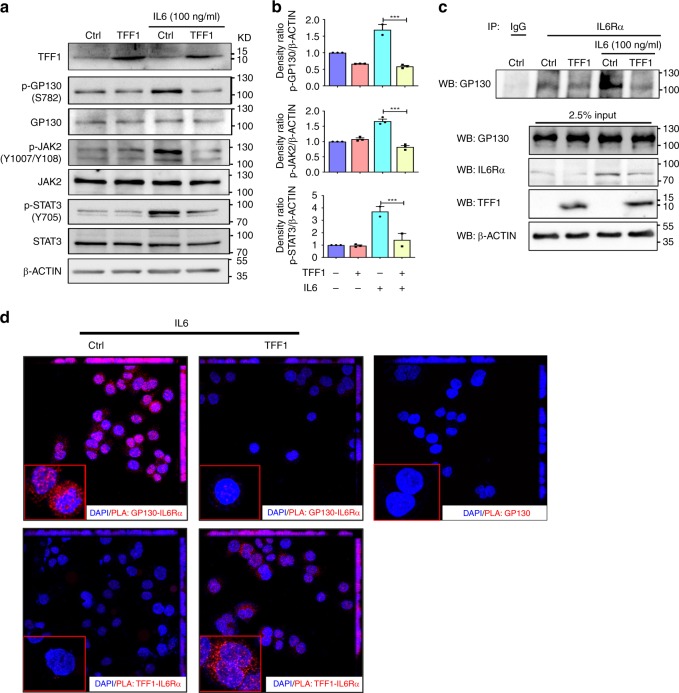
TFF1 negatively regulates IL6-induced STAT3 activation through GP130-IL6Rα axis. **a** Western blot analysis using AGS cell lines infected with control or TFF1 adenovirus. After stimulation with IL6 (100 ng mL^−1^) for 30 min, TFF1 expressing cells showed a significant decrease of p-STAT3 (Y705), p-GP130 (S782), and p-JAK2 (Y1007/Y1008) protein levels as compared to control cells. **b** The relative intensity ratio of p-JAK2/β-Actin, p-GP130/β-Actin, and p-STAT3/β-Actin were calculated by Image-lab software from BioRad. The results are expressed as mean ± SEM of at least three independent experiments. ^***^P < 0.001 by two-tailed Student’s *t* test. **c** Immunoprecipitation and Western blot analysis following IL6Rα pulldown using AGS cells infected with TFF1 or control adenoviruses (5 MOI), with or without treatment with IL6 (100 ng mL^−1^) for 30 min. The first lane exhibits AGS control following immunoprecipitation with mouse IgG control antibody. All immunoprecipitations and their corresponding input samples were subjected to immunoblotting with rabbit polyclonal antibody against GP130 and IL6Rα. The expression of TFF1 and equal amounts of protein loading were confirmed in the input samples. **d** Punctate proximity ligation assay (SOURCE) was performed in accordance with supplier’s instructions in AGS cells infected with control or TFF1 adenovirus. The presence of red signals indicates positive ligation, indicative of interaction. Using IL6Rα and GP130 antibodies, the results indicated the presence of IL6Rα–GP130 interaction (red signals), following stimulation with IL6 in control cells (Ctrl). This interaction was not detected in AGS cells expressing TFF1 (upper left two panels). The lower panel displays immunofluorescence following the use of TFF1 and IL6Rα antibodies and demonstrates an interaction between TFF1 and IL6Rα (red signals), (lower right two panels). As a negative control for PLA background reaction, control cells were stimulated with IL6 and probed with a single antibody for GP130 (right single panel). Maximum intensity projection is presented in the upper and right side of each image

Our results, thus far, indicate that recombinant TFF1 protein can suppress IL6-mediated activation of STAT3. IL6-mediated activation of STAT3 involves activation of IL6Rα, which mediates activation of IL6 signal transducer (IL6ST, also known as GP130). This leads to phosphorylation and activation of Janus Kinase 2 (JAK2), phosphorylation and dimerization of STAT3, followed by STAT3 nuclear translocation^22,23^. Therefore, we explored if TFF1 interferes with the formation and activation of the IL6–IL6Rα–GP130 complex in gastric cancer. Using AGS and STKM2 gastric cancer cell lines, we confirmed by Western blot analysis the activation and increase of phospho-STAT3 (Y705) in control cells after IL6 stimulation (100 ng mL^−1^) for 30 min (Fig. 7a, b and Supplementary Fig. 9a, b). This increase was significantly abolished after reconstitution of TFF1 (Fig. 7a, b and Supplementary Fig. 9a, b, *P* < 0.001). As expected, the phospho-GP130 (S782) and the phospho-JAK2 (Y1007/Y1008) were increased in AGS and STKM2 control cells after IL6 stimulation. This increase was significantly reduced after reconstitution of TFF1, as demonstrated by the density ratio of the phospho-GP130 (S782) and phospho-JAK2 (Y1007/Y1008) (Fig. 7a, b and Supplementary Fig. 9a, b, *P* < 0.001). Based on these data, we hypothesized that TFF1 may interfere with the formation of IL6Rα–GP130–JAK2 complex and recruitment of STAT3, following IL-6 stimulation. To confirm this hypothesis, we tested the binding of IL6Rα and GP130 proteins by performing an IL6Rα immunoprecipitation assay followed by immunoblot analysis of GP130 in conditions of IL6 stimulation with or without TFF1 reconstitution in AGS and STKM2 cells. The results indicated the presence of high levels of GP130 in immunoprecipitated protein in the control cells, following IL6 stimulation. GP130 was almost undetectable in AGS and STKM2 cells expressing TFF1 (Fig. 7c and Supplementary Fig. 9c). These findings suggest that TFF1 can suppress or interfere with the complex formation of IL6Rα and GP130, following IL6 stimulation.

### TFF1–IL6Rα binding inhibits IL6–IL6Rα–GP130 complex formation

To validate the immunoprecipitation assay and confirm the inhibitory effect of TFF1 on the IL6–IL6Rα–GP130 complex formation, we performed an in situ proximal ligation assay (PLA), a developing method in bioanalysis to detect in situ interactions or co-localization of proteins in proximity of ≈16 nm^24^, using fluorescent microscopy. The PLA was performed using antibodies specific against IL6Rα and GP130, as described in the “Methods” section, on AGS cells expressing TFF1 via the adenoviral expression system. Following IL6 stimulation of AGS expressing control adenovirus, we detected an increase in the ligation signals of IL6Rα and the GP130 (red fluorescence), indicating a positive interaction between IL6Rα and GP130 (Fig. 7d). In contrast, the reconstitution of TFF1 abrogated ligation signals of IL6Rα and GP130 following IL6 stimulation, suggesting loss of IL6Rα and GP130 interaction after TFF1 reconstitution (Fig. 7d). These data indicate that TFF1 interferes with binding of IL6α and GP130. Therefore, we addressed the question of whether TFF1 can directly interact with IL6Rα and interfere with IL6-mediated IL6Rα–GP130 complex formation. PLA was performed using antibodies specific against TFF1 and IL6Rα. In AGS cells stably expressing TFF1, we detected positive ligation signals (red dots) between TFF1 and IL6Rα, indicating that TFF1 and IL6Rα are in close proximity (Fig. 7d). AGS cells expressing empty vector were used as a control.

**Fig. 8:**
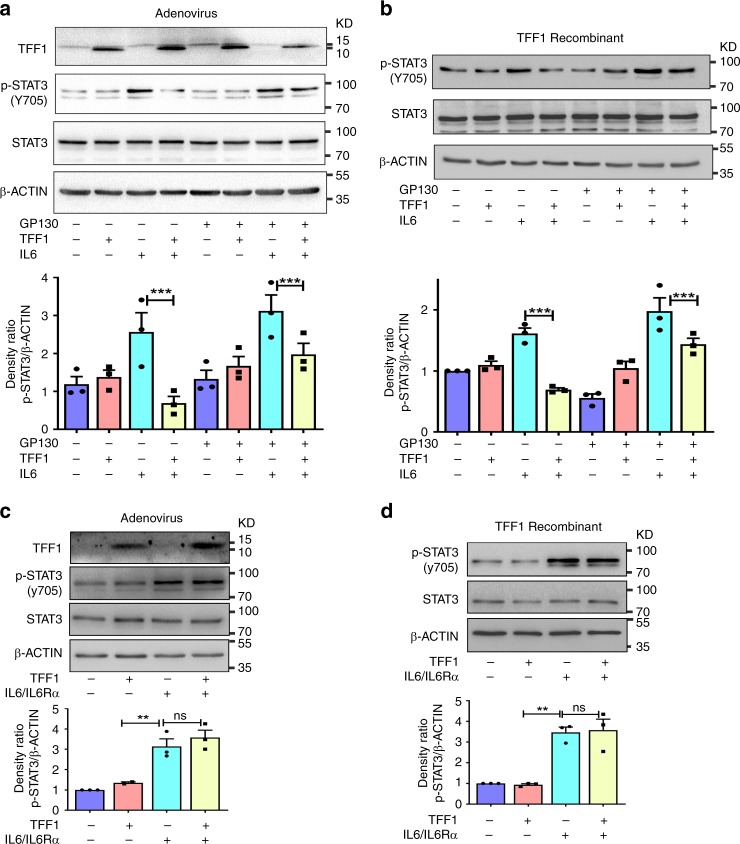
IL6–IL6Rα soluble complex antagonizes TFF1-mediated suppression of STAT3 activation. **a** AGS cells were infected with control or TFF1 adenoviruses (5 MOI), transfected or not with GP130 expression plasmid (500 ng mL^−1^) and stimulated or not with IL6 (100 ng mL^−1^). AGS expressing TFF1 showed a decrease of p-STAT3 (Y705) after GP130 transfection and IL6 stimulation. **b** AGS cells were transfected or not with GP130 expression plasmid (500 ng mL^−1^) and treated with recombinant TFF1 (400 ng mL^−1^), then stimulated or not with IL6 (100 ng mL^−1^). TFF1 treated cells showed a decrease of p-STAT3 (Y705) after GP130 transfection and IL6 stimulation. **c** AGS cells were infected with control or TFF1 adenoviruses (5 MOI) and treated or not with IL6–IL6Rα soluble complex (10 ng mL^−^^1^). AGS-expressing TFF1 showed no effect of p-STAT3 (Y705) after IL6–IL6Rα treatment as compared to control. **d** AGS cells were treated or not with TFF1 recombinant protein (400 ng mL^−1^) and, or IL6–IL6Rα soluble complex protein (10 ng mL^−^^1^). AGS cells treated with TFF1 and IL6–IL6Rα showed no effect of p-STAT3 (Y705) as compared to control cells treated with IL6–IL6Rα alone. The relative intensity ratio p-STAT3/β-Actin were calculated by the Image-lab software from BioRad, and presented under each blot. The results are expressed as mean ± SEM of at least 3 independent experiments; ***P < 0.001 by two-tailed Student’s *t* test, and *P* > 0.05 is considered nonsignificant (ns)

Next, we investigated if TFF1 reconstitution and interaction with IL6Rα can interfere with STAT3 activation following overexpression of GP130 and stimulation with IL6. To perform this rescue experiment, AGS cells were infected with TFF1 adenoviruses for 24 h, followed by transient transfection with GP130. The following day, cells were stimulated with IL6 for 30 min, and collected for Western blot analysis. The reconstitution of TFF1 by adenoviral particles was sufficient to decrease the levels of phospho-STAT3 (Y705), following IL6 stimulation, in all experimental conditions, including overexpression of GP130, as compared to control (Fig. 8a, *P* < 0.001). Similar results were obtained using TFF1 recombinant protein (Fig. 8b). Collectively, our data demonstrate the suppressive effect of TFF1 on STAT3 via interfering with the IL6–IL6Rα–GP130 complex formation.

To gain more insight into the role of TFF1, we investigated whether TFF1 can suppress the stimulatory effect of an already formed IL6–IL6Rα complex or not. Therefore, we treated cells with soluble IL6–IL6Rα complex recombinant protein (10 ng mL^−1^) for 12 h, following reconstitution of TFF1 expression with adenoviral particles or recombinant protein, as compared to controls. Western blot analysis showed a significant increase of phospho-STAT3 in control cells after treatment with IL6–IL6Rα soluble complex, as compared to non-treated AGS control cells. The reconstitution of TFF1 in these conditions had no significant effects on inhibiting phospho-STAT3 levels (Fig. 8c, d). These results indicate that the soluble IL6–IL6Ra complex was able to bind GP130 and activate phospho-STAT3 irrespective of TFF1 levels. Based on the PLA results and these findings, it is more likely that TFF1 binds to IL6Rα to interfere with binding of the ligand, IL6.

To confirm that TFF1 can indeed interfere with IL6–IL6Rα, we performed a quantitative ELISA assay (R&D systems) and checked the levels of soluble IL6–IL6Rα complex formation, after stimulation with IL6. AGS cells were infected with TFF1 or control adenoviruses. After 48 h, cells were stimulated with IL6 for 3 h and supernatant media was collected for analysis. The quantification of ELISA data showed a significant decrease of the recombinant human IL6–ILRα in AGS cells expressing TFF1, as compared to control cells, following IL6 stimulation (Supplementary Fig. 10, *P* < 0.01). Taken together, our results imply that TFF1 inhibits IL6-mediated STAT3 activation through binding to IL6Rα and interfering with the IL6–IL6Rα–GP130 complex formation.

### TFF1 and STAT3 expression patterns in human tissue microarrays

**Fig. 9:**
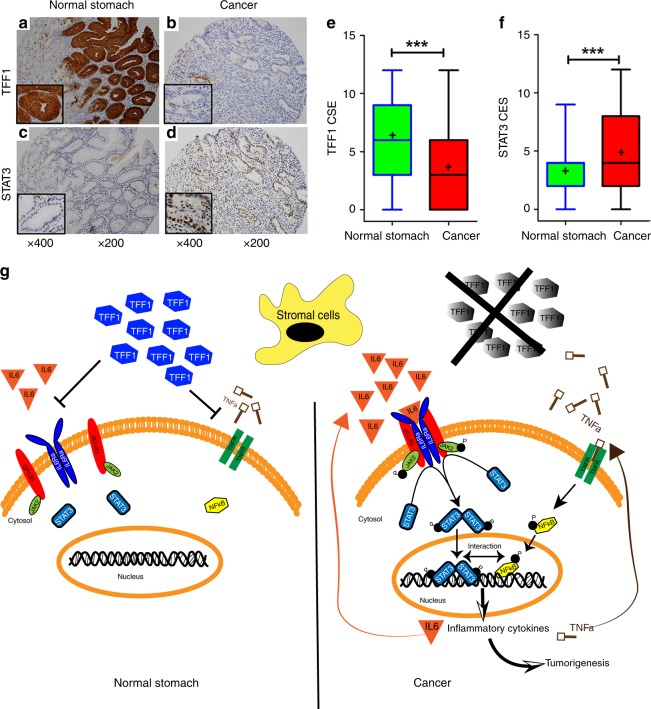
Human gastric cancer tissues demonstrate nuclear localization of STAT3 and loss of TFF1. **a**–**f** Representative immunohistochemical (IHC) staining images of TFF1 (upper panels **a** and **b**) and STAT3 (lower panels **c** and **d**) using human gastric cancer tissue microarrays that contained 108 cancer samples with their matching adjacent histologically normal stomach, original magnification is at ×200 and insets are at ×400. **e**, **f** Summary graphs of the IHC staining of TFF1 (**e**) and nuclear STAT3 (**f**) results using box-and-whisker blots to depict the smallest value, lower quartile, median, upper quartile, and largest value, (+) indicate the mean. **g** Schematic cartoon representing the role of trefoil factor 1 (TFF1) in regulating inflammatory signaling in gastric epithelial cells. Gastric cancer is associated with inflammation mediated by the release of cytokines such IL6 and TNFα from stromal and epithelial cells inducing activation of STAT3 and NFκB transcriptional factors. Loss of TFF1 promotes IL-6-mediated IL6Rα-GP130 complex formation, recruitment and phosphorylation of JAK2, with subsequent phosphorylation and nuclear localization of STAT3 to activate STAT3 transcription targets. The role of TFF1 in regulating NFκB has been described before^16^

Using IHC on gastric cancer tissue microarrays, we evaluated the protein expression of TFF1 and STAT3 in human gastric cancer and normal tissue samples. The TFF1 protein expression levels in gastric cancer were significantly lower than normal gastric tissues (Fig. 9a, b, e, *P* < 0.001). On the other hand, gastric cancer tissues showed high expression levels of nuclear STAT3, as compared to normal gastric tissues (Fig. 9c, d, f, *P* < 0.001). The IHC analysis results of TFF1 and STAT3 are summarized in Fig. 7e, f, respectively. A schematic graph summarizing the effects of TFF1 loss on gastric tumourigenesis is shown in Fig. 9g.

## Discussion

In this study, we provide multiple lines of evidence, using in vitro and in vivo models, supporting the role of TFF1 in suppressing STAT3 activity via interfering with the formation of the IL6–IL6Rα–GP130 signaling complex. This finding further supports the role of TFF1 as an anti-inflammatory peptide with tumor suppressor functions in gastric mucosa.

TFF1 is a small secreted protein (6.5 kDa), predominantly expressed in normal gastric mucosa, that plays critical roles in keeping the normal mucosa barrier integrity^25^. It also promotes mucosal repair and healing after injury^14^. For example, TFF1 binding to MUC5AC is essential to form an intact protective mucous layer^26^. Loss of TFF1 is associated with aberrant expression of CLDN7, a tight junction protein critical for barrier function^27,28^. Loss of barrier function is an early step in cellular transformation^29^. Several recent reports indicate that loss of TFF1 expression and its mucosal protective capacity mediate activation of oncogenic pathways, suggesting that TFF1 has tumor suppressor functions in gastric mucosa^15,16,21^. We previously reported that the loss of TFF1 in human and mice activated oncogenic pathways and promoted tumourigenesis in the antropyloric area of gastric tissues^16,30,31^. In this study, we demonstrate phosphorylation and nuclear localization of STAT3 in non-neoplastic gastric mucosa of the *Tff1*-Knockout mouse. Activation of STAT3 is an early step in carcinogenesis that maintains and promotes a protumourigenic microenvironment^32,33^. Of note, a recent integrated molecular bioinformatics study suggested activation of STAT3 transcription network in mouse and human gastric tumourigenesis^17^, a finding that is further supported by GSEA analysis of STAT3 target genes. In fact, activation of STAT3 in gastric tumourigenesis plays a critical role in tumourigenesis^34^ and resistance to therapeutics^35^. Our findings suggest that silencing TFF1 could be an early event that triggers the activation of STAT3 in gastric mucosa during gastric tumourigenesis. Loss of TFF1 is a frequent finding in human gastric cancer that occurs via a number of molecular alterations such as LOH^18^, promoter methylation^18,36^, and transcription suppression^37^. The fact that TFF1 is silenced in the majority of human gastric cancers by genetic, epigenetic, and transcription mechanisms highlights the importance of our results and denotes a previously unknown protective function of TFF1 against the oncogenic activation of STAT3 in gastric cancer. Earlier studies have shown that TFF1 protects the gastric mucosa integrity and barrier functions with suppression of oncogenic activation of β-catenin and NF-kB^16,21^, known to play important roles in gastric carcinogenesis. Taken together with our results, the current study supports the tumor suppressive role of TFF1, where its silencing triggers loss of mucosal integrity with activation of a plethora of signaling events, including STAT3, that acts concertedly to promote tumourigenesis.

The activation of STAT3 transcription activity induces expression of a wide range of target genes that promote key pro-oncogenic cellular functions such as inflammation, proliferation, survival, invasion, and angiogenesis^7,38^. Our results demonstrated nuclear localization of STAT3, increased transcription activity, and upregulation of several STAT3 target genes such as *IL17A, c-MYC*, *VEGF*, *BCL2*, and *BIRC5*, in mouse and human models of loss of TFF1. Collectively, these genes play an important role in regulating cellular functions in cancer development and progression^39,40^. For example, *BIRC5* (also known as *Survivin*) and *BCL2* are anti-apoptotic genes that promote cancer cell survival, whereas *IL17A*, *IL23* and *IL6* play important roles in promoting pro-inflammatory oncogenic signaling^41,42^. Of note, the expression of these genes was detected in non-neoplastic gastric mucosa of the TFF1-KO mice, suggesting a direct link between TFF1 loss and activation of STAT3. A progressive increase in the expression level of these STAT3 target genes was detected with advancing ages and the development of neoplastic lesions in the TFF1-KO mice, confirming an important role of STAT3 in initiation and progression of neoplastic gastric lesions. The reconstitution of TFF1 levels in TFF1-KO gastric gland organoids and human gastric cancer cells, significantly decreased the phosphorylation and nuclear localization of STAT3 with reduced expression of several known STAT3 target genes, further establishing a link between TFF1 and STAT3.

The IL-6 family cytokines is a group of cytokines including IL6, IL11, IL27, and several others that form a complex with signaling receptor subunit GP130 for activation of STAT3^43^. In this study, we used IL6 as an example and demonstrated that TFF1 can suppress IL6-mediated activation of STAT3. Activation of STAT3 by IL6 cytokine requires its binding to IL6Rα, also known as GP80, to form a heterodimeric complex of IL6–IL6Rα that associates with GP130, resulting in a hexameric structure inducing activation and phosphorylation of GP130^44^. This activation promotes phosphorylation of JAK kinases at Y1007/1008 with subsequent phosphorylation and nuclear translocation, and transcriptional activation of STAT3^10,11^. Co-immunoprecipitation results indicated that TFF1 interfered with the interaction between IL6Rα and GP130. This notion is also supported by additional pieces of evidence, such as the PLA assay, showing that TFF1 can abrogate the interaction between IL6Rα and GP130, following IL6 stimulation. The results indicated that TFF1 interacted with IL6Rα, thereby interfering with IL6-mediated formation of IL6–IL6Rα heterodimer complex and activation of the IL6–IL6Rα–GP130 signaling complex. In fact, this assumption is augmented by failure of TFF1 to interfere with the activation of STAT3 when an intact soluble human complex IL6–IL6Rα was provided. Together, we concluded that TFF1 interacted with IL6Rα to inhibit the formation of the IL6–IL6Rα/GP130 complex, the first step in a cascade of signaling events leading to the activation of STAT3. Nevertheless, a recent study showed that aberrant activation of STAT3 suppressed TFF1 expression partially through epigenetic silencing of *GATA6* gene^45^, suggesting the presence of a negative regulatory feedback loop between TFF1 and STAT3 in cancer cells; not investigated in our study.

Several recent studies suggest a collaboration and cross-talk between STAT3 and NF-κB to promote the development of several cancers, including gastric cancer^46^. In this context, we have previously shown that loss of TFF1 mediates activation of NF-κB. NF-κB and STAT3 are two main pro-inflammatory signaling pathways that undoubtedly play major roles in inflammation-driven cancers where their-co-activation has a synergistic effect on expression of oncogenic and pro-inflammatory transcription targets^47,48^ (Fig. 9). TFF1 inhibits TNF-α-mediated NF-κB activation through TNF receptor 1 (TNFR1)-IκB kinase (IKK) pathway^16^. Based on our findings, it is plausible to suggest that TFF1 is a major anti-inflammatory peptide that may have potent, suppressive effects against inflammation-driven development and progression of gastric cancer. The fact that TFF1 can interact with TNFR1 and IL6Rα, also raises interesting and important questions regarding the extent of extracellular functions of TFF1. Although beyond the scope of the current study, it remains to be investigated whether TFF1 interacts with other receptors involved in tumourigenesis.

## Conclusion

Our results suggest that TFF1 plays an essential role in protecting against IL6-mediated activation of STAT3 by interfering with the formation of the IL6–IL6Rα–GP130 complex. Silencing TFF1 with loss of its mucosal protective functions is possibly a crucial early step that induces activation of pro-inflammatory oncogenic signaling pathways, such as NF-kB and STAT3 in gastric tumourigenesis.

## Methods

### Animals

C57BL/6J/129/Svj TFF1-knockout mice and normal TFF1*-*wild type from the same background^15^ were used in this study. In total, tissue samples were collected from 126 TFF1-knockout and 44 TFF1-wild type mice. All animals were approved by the Institutional Animal Care and Use Committees at Vanderbilt University Medical Center and University of Miami. Following euthanasia, animals were dissected through a midline incision of the abdomen. Classification and grading of the gastric tissues were performed by our pathologists (M.B.P. and M.K.W.).

### Primary gastric epithelial cells and 3D organoid cultures

Short-term cultures of primary gastric epithelial cells were prepared following our standard protocol^16^. 3D organoid tissue cultures were prepared based on the protocol by Barker et al.^49^. Stomachs were removed from 4 to 8-week-old TFF1-WT and TFF1-KO mice. Stomachs were opened, washed with HBSS, and the gastric antrum was cut and incubated in 5 ml of 5 mM EDTA for 2 h at 4 °C with gentle shaking. The EDTA was replaced with 5 ml chelation buffer and tissue was shaken vigorously for approximately 2 min to dissociate glands. Dissociated glands were passed through a 70 µm nylon mesh cell strainer (Becton Dickson, Franklin Lakes, NJ) to remove muscular layers of the stomach, then centrifuged at 150*g* for 5 min. The pelleted glands were embedded in an extracellular matrix hydrogel (Trevigen, Inc., Gaithersburg, MD) supplemented with IntestiCult Organoid Growth Medium (StemCell Technologies, Vancouver), Noggin (100 ng mL^−1^), Wnt-3A (100 ng mL^−1^), epidermal growth factor (50 ng mL^−1^), fibroblast growth factor 10 (100 ng mL^−1^) (Peprotech Inc., Rocky Hill, NY), R-spondin (1 µg mL^−1^) (Antibody & Protein Resource at Vanderbilt VUMC core), and gastrin (10 nM) (Sigma, St. Louis, MO). Glands matured into organoids by the third day, and were passaged every 12 days. Organoids were removed from Matrigel using gentle cell dissociation reagent (StemCell Technologies, Vancouver), and processed for standard formalin fixed embedded tissues.

### Reconstitution of TFF1 expression

AGS cells were obtained from American Tissue Culture Collection (ATCC, Manassas, VA, USA). STKM2 cells were a generous gift from Dr. Alexander Zaika (University of Miami, FL, USA). Cells were cultured in Ham’s F-12 supplemented with 10% fetal bovine serum (Invitrogen Life Technologies, Carlsbad, CA, USA), and incubated at 37 °C in an atmosphere containing 5% CO_2_. In order to reconstitute the expression of TFF1, we used the transient adenovirus system and the stable Lenti-X^TM^ TetOne^TM^ Inducible Expression System (Clontech). For the adenovirus system, the TFF1 coding sequence from pcDNA3.1-TFF1 plasmid was subcloned into the adenoviral shuttle vector (pACCMV). The recombinant adenovirus-expressing TFF1 was generated by co-transfecting HEK-293 cells with the shuttle and backbone adenoviral (pJM17) plasmids using the Calcium Phosphate Transfection kit (Applied Biological Materials). The adenovirus vector (pACCMV) was used as negative control. After infection, the AGS and STKM2 cells were analyzed for the expression of *TFF1* by qRT-PCR^20^. For generating stable doxycycline inducible clones of *TFF1* insert under the *TRE3G* promoter in AGS cells, the coding sequence of TFF1 was cloned into Bam HI and EcoRI enzyme digested TetOne vector plasmid (Clontech), the resulting TetOne-TFF1 plasmids with appropriate inserts were verified by PCR, restriction enzyme digestions and DNA sequencing. AGS cells were transfected with TetOne-TFF1 and selected with hygromycin (0.2 μg ml^−1^, Sigma) for 2 weeks. The surviving cells were expanded and screened for TFF1 expression after doxycycline (500 ng mL^−1^) treatment for 48 h. The TFF1 expressing cells were then seeded in 96-well plates at one single-cell per well. For conditioned media, we used pcDNA-TFF1-AGS and pcDNA-AGS stable cell lines. The human TFF1 coding sequence was amplified using PCR and cloned in-frame into pcDNA3.1 mammalian expression vector (Invitrogen), AGS cells were transfected with pcDNA3.1-TFF1 or empty vector (control) using Fugene-6 (Roche Applied Science) following the manufacturer’s protocols. Stable transfectants were selected using 0.5 mg mL^−1^ G418 (Invitrogen) and TFF1 was analyzed by qRT-PCR.

### Human tissue microarrays and IHC

Tissues from gastric cancer patients with adjacent histologically normal samples were stained with H&E and examined by our pathologists. The adenocarcinomas collected ranged from well-differentiated to poorly differentiated, stages II–IV, with a mix of intestinal and diffuse-type tumors. Representative regions were selected for inclusion in a tissue microarray using a manual tissue array instrument (Beecher Instruments, Silver Spring, MD, USA). Tissue microarrays containing cores from 108 paraffin-embedded gastric cancer tissue samples with matching normal tissues were available for immunohistochemical (IHC) analysis. All tissue samples were collected, coded and de-identified in accordance with Vanderbilt University Medical Center Institutional Review Board-approved protocols. Sections of 5 µm were transferred to poly lysine-coated slides (SuperFrostPlus, Menzel-Glaser, Braunschweig, Germany). Slides were placed on the Leica Bond Max IHC stainer, and all steps besides dehydration, clearing and cover slipping are performed. Slides were deparaffinized and heat-induced antigen retrieval was performed on the Bond Max using their Epitope Retrieval solution for 15–20 min. Next, slides were incubated with anti-STAT3 (cat#ab76315, Abcam, Cambridge, MA) or anti-TFF1 (cat# TA322883, Origene, Rockville, MD) for one h at a 1:100 dilution, and the Bond Polymer Refine Detection System was used for visualization. Slides were then dehydrated, cleared and cover slipped. For scoring IHC staining intensity (I), the nuclear and cytosolic intensity of staining is graded as 0 (negative), 1 (weak), 2 (moderate), and 3 (strong). The frequency (*F*) is graded from 0 to 4 by the percentage of positive cells as follows: grade 0, <3%; grade 1, 3–25%; grade 2, 25–50%; grade 3, 50–75%; grade 4, more than 75%. The index score was calculated as a product of I × F with a range from 0 to 12.

### Luciferase reporter assay

The STAT3–Luciferase reporter vector (Addgene, Cambridge, MA) was used to measure the transcriptional activity of STAT3^50^. For adenovirus system, gastric cancer cell lines, AGS and STKM2, were infected with TFF1 or Control adenovirus (5 MOI). The next day, cells were transiently transfected with 500 ng of STAT3–Luc and 250 ng of β-galactosidase as a control plasmid for transfection using FuGENE 6 according to the manufacturer’s instructions (Roche Applied Science). After 48 h, cells were treated with IL6 (100 ng ml^−1^) (GenScript USA Inc.) for 3 h, and luciferase and β-galactosidase activities were measured. The firefly luciferase activity was normalized to β-galactosidase activity. For recombinant TFF1, cells were transfected with STAT3–Luc and β-galactosidase. The next day, cells were treated with TFF1 recombinant protein (400 ng mL^−1^). After 24 h, cells were stimulated with IL6 for 3 h, and luciferase and β-galactosidase activities were measured.

### Western blot analysis

Cell lysates were prepared in RIPA buffer containing Halt Protease Inhibitor Cocktail and Halt Phosphatase Inhibitor Cocktail (Pierce, Rockford, IL, USA), and were centrifuged at 3500 r.p.m. for 10 min at 4 °C. Protein concentration was measured using Bio-Rad Protein Assay (Bio-Rad Laboratories, Hercules, CA, USA). Proteins (10–15 μg) from each sample were subjected to sodium dodecyl sulphate (SDS)/polyacrylamide gel electrophoresis (PAGE) and transferred onto nitrocellulose membranes. Target proteins were detected using specific antibodies against phospho-STAT3 (Y705) (cat# 9145S), phospho-JAK2 (Y1007/Y1008) (cat# 3771), STAT3 (cat# 12640), JAK2 (cat# 3230 S), and β-actin (cat#3700) (purchased from Cell Signaling Technology, Beverly, MA). Antibodies against phospho-GP130 (Ser782) (cat#sc-377572) and GP130 (cat# sc-376280) were obtained from Santa Cruz Biotechnology, Inc. (Santa Cruz, CA). For nuclear and cytoplasmic protein fractions, we used NE-PER Nuclear and Cytoplasmic Extraction Reagents (Pierce Biotechnology Inc.) following the manufacturer’s instructions. The cytoplasmic and nuclear protein fractions were normalized to Anti-NaKATPase (cat# ab76020) (Abcam, Cambridge, MA) and lamin B (cat# sc-374015) (Santa Cruz, CA), respectively. All antibodies were used at 1:1000 dillution.

### Immunoprecipitation

AGS and STKM2 cells were washed with cold phosphate-buffered saline, scraped and resuspended in 1 ml of cell lysis buffer from an MCL1–1KT Mammalian Cell Lysis Kit (Sigma-Aldrich, Milwaukee, WI). Cells were rocked for 15 min at 4 °C followed by sonication for 10 s for a total of 3 times; lysates were centrifuged for 10 min at 12,000*g* at 4 °C. Immunoprecipitation was performed using Dynabeads Protein G (Dynal, Invitrogen Life Sciences, Carlsbad, CA) according to the manufacturer’s instructions. IL6Rα antibody (Santa Cruz Biotechnology) was cross-linked to Dynabeads Protein G. The cell lysate was added to the cross-linked beads and incubated for 2 h with rocking. The Dynabeads were then pelleted using a magnet and washed three times with washing buffer. Captured protein was eluted from the beads by adding 40 μl of 2× protein-loading buffer to each sample and boiling for 10 min. Samples were resolved by SDS/PAGE and subjected to Western blotting. The membrane was incubated with total GP130 antibody (Santa Cruz Biotechnology) overnight.

### ChIP assay

AGS cells were fixed with 1% formaldehyde final concentration (Sigma-Aldrich) and chromatin fragmentation was done by sonication on ice for four cycles (30 s “ON”, 30 s “OFF” at 40% amplitude) to yield an average length of 235 bp. ChIP assay was performed using the Zymo-Spin ChIP Kit (Irvine, California, USA) following the manufacturer’s protocol. Briefly, the supernatants of the fragmented lysates were diluted tenfold with chromatin dilution buffer. Chromatin solutions were immunoprecipitated with STAT3 antibody from cell signaling at 4 °C overnight. ZymoMag Protein A beads were added to the lysate to isolate the antibody-bound complexes. The eluate was reverse cross-linked by heating at 65 °C for 30 min. Samples were then treated with proteinase K for 90 min at 65 °C to digest the proteins that were immunoprecipitated. The final eluate is purified DNA, which was analyzed by quantitative real-time PCR for STAT3 binding to the promoter sequences of *VEGF* and *IL6* target genes, and normalized to the input. The primer sequences were VEGFA-CHIP-F: 5′-AGACTCCACAGTGCATACGTG-3′ and VEGFA-CHIP-R: 5′-AGTGTGTCCCTCTGACAATG-3′; IL6-CHIP-F: 5′-GTT GTG TCT TGC CAT GCT AA A G-3′ and IL6-CHIP-R: 5′-AGA ATG AGC CTC AGA CAT CTC C-3′. The control primers were designed 500 base pair upstream of STAT3 binding site on the *IL6* promoter; the sequences were CTRL-F: 5′-GAG AAA GGA GGT GGG TAG GC-3′ and CTRL-R: 5′- AAA AGG AAG CCC TGA GAA GC-3′.

### Proximity ligation assay

To determine protein–protein interaction at the cellular level, we performed proximity ligation assay using Duolink in Situ Red Starter Kit Mouse/Rabbit (Sigma-Aldrich). AGS gastric cancer cells infected with control or TFF1 adenovirus were seeded in 8-chamber slides (500 cells/chamber). Next day, cells were stimulated with IL6 (100 ng mL^−1^) for 3 h, and fixed with fresh 4% paraformaldehyde solution for 15 min at room temperature. Cells were then permeabilized with 1% Triton X (Sigma, St. Louis, MO) for 2 min on ice, followed by incubation in a humidified chamber in 10% normal goat serum blocking solution (Zymed Laboratories, Carlsbad, CA, USA) for 20 min at room temperature. We determined the interaction between the GP130 and IL6Rα proteins by using specific primary antibodies, mouse anti IL6Rα (Cat#sc-373708, Santa Cruz Biotechnology) and rabbit anti GP130 (Santa Cruz Biotechnology). We also determined the interaction between IL6Rα and TFF1 proteins by using mouse anti IL6Rα and rabbit anti TFF1 antibodies (cat#TA322883, Origene, Rockville, MD). Immunofluorescence was performed following the supplier’s instructions. Briefly, cells were incubated with a combination of two antibodies, one from mouse and one from rabbit donors, diluted in antibody diluent solution (1:100) overnight at 4 °C. The following day, primary antibodies were removed from the chamber slide. The slide was washed once, following the supplier’s protocol, and the secondary antibodies conjugated with oligonucleotides (Duolink In Situ PLA Probe Anti-Mouse Minus and Duolink In Situ PLA Probe Anti Rabbit Plus; Sigma-Aldrich) were added and incubated in a preheated humidity chamber for 1 h at 37 °C. The probes were removed and the slide was washed twice for 5 min under gentle agitation. The ligation–ligase solution was added to each sample and incubated in a preheated humidity chamber for 30 min at 37 °C. For amplification, the slide was washed twice for 2 min and the amplification polymerase solution was added and incubated for 100 min in a preheated humidity chamber at 37 °C. Finally, the slides were washed, dried, and an in situ mounting medium with DAPI (Sigma) was applied. Nonspecific signals were assessed by using single primary antibody staining for GP130, as a control. Fluorescence was detected using fluorescence confocal microscopy (Zeiss LSM 880).

### Statistics

Using the GraphPad Prism software, a t test was used to compare the statistical difference between two groups and a one way ANOVA Newman–Keuls Multiple Comparison Test was used to compare the differences between three groups or more. The differences were considered statistically significant when the *P* value was ≤0.05.

The methods that describe immunofluorescence, quantitative RT-PCR, rescue of secreted TFF1 protein in 3D organoid cultures are included in the online Supplementary Methods.

### Reporting summary

Further information on research design is available in the Nature Research Reporting Summary linked to this article.

## Supplementary information

Supplementary information

Reporting Summary

## Data Availability

The data that support the findings of this study are available from the corresponding author upon reasonable request.
