# Supplementary material for: Activation of STAT3 signaling is mediated by TFF1 silencing in gastric neoplasia
Source: Nat Commun. 2019 Jul 10;10:3039. doi: 10.1038/s41467-019-11011-4 (PMC6620282; doi:10.1038/s41467-019-11011-4)

Supplementary methods

Immunofluorescence assay

Mouse tissues and 3D organoid cultures from TFF1-WT and TFF1-KO mice were fixed in 10 % formalin, embedded in paraffin, and blocks were cut to 5 µm sections on glass slides. Tissue sections were deparaffinized and heated in a pressure cooker for 12 min in 1xTE buffer to perform antigen retrieval. Sections were blocked with 1xPBS containing 5% bovine serum albumin for 1h at room temperature. The sections were incubated with primary antibodies p-STAT3 (Y705) (Abcam, Cambridge, MA) and ZO1 (ZYMED Laboratories, Invitrogen) diluted in blocking buffer (1:200) overnight at 4°C. Next day, sections were washed and incubated with secondary antibodies goat anti-rabbit IgG conjugated to fluorophore Alexa Fluor 488 and goat anti-mouse IgG conjugated to Alexa Fluor 568 (Invitrogen) diluted in blocking buffer (1:500) for 1h at room temperature. Sections were washed three times and mounted with Vectashield/DAPI (Vector Laboratories, Burlingame, CA, USA). Sections were imaged on a Zeiss confocal microscope, using Zeiss ZEN software (Carl Zeiss Microscopy, Thornwood, NY).

Primary ex-vivo mouse gastric epithelial cells were prepared as described above. AGS gastric cancer cells expressing TFF1 or empty pcDNA vector were plated in 8-well chambers. Cells were washed with PBS and fixed with fresh 4% paraformaldehyde solution for 15 min at room temperature. Cells were then washed twice with PBS and incubated in 10% normal goat serum blocking solution (Zymed Laboratories, Carlsbad, CA, USA) in a humidified chamber for 20 min at room temperature. Cells were incubated in p-STAT3 (Y705) (Abcam, Cambridge, MA) primary antibody diluted in PBS (1:400) for 2h at room temperature in a humidified chamber. Cells were washed three times with PBS and incubated in a humidified chamber with a goat anti-rabbit IgG secondary antibody conjugated to Alexa Fluor 488 (1:500, Invitrogen) for 45 min at room temperature. Cells were washed in PBS, mounted with Vectashield/DAPI (Vector Laboratories, Burlingame, CA, USA) and visualized using an Olympus BX51 fluorescence microscope (Olympus Co., Tokyo, Japan). At least 200 cells were counted from each experiment. Total cell number was measured with automatic particle counting in ImageJ software (http://www.uhnresearch.ca/facilities/wcif/imagej/), after setting an automatic threshold range.

The image was transformed into a binary image and the total number of cells in each field were counted using watershed separation. The percentage of nuclear STAT3 positive cells was calculated as the number of cells showing green nuclear immunostaining divided by the total cell number showing DAPI (blue) nuclear staining × 100.

Quantitative real-time RT-PCR (qRT-PCR)

RNA was isolated using the RNeasy Mini Kit (Qiagen, Valencia, CA, USA), and single-stranded cDNA was subsequently synthesized using the Advantage RT-for-PCR Kit (Clontech Laboratories Inc., Palo Alto, CA, USA). Genes specific for mouse and human primers were designed using the online software, Primer 3 (http://frodo.wi.mit.edu/cgi-bin/primer3/primer3_www.cgi). Forward and reverse primers were designed to span two different exons for each gene (mouse: *Vegf, c-Myc, Birc5, Il17a, Il23, Il11, Il6, Ccl2, Ccl3, Bcl2* and *Hprt;* Human: *VEGF, C-MYC, CXCL10, IL17A, TFF1 and HPRT*). All primers were purchased from Integrated DNA Technologies (Coralville, IA, USA) (Supplementary Table S1). qRT-PCR was performed using the CFX96 Real-Time PCR Detection System (Bio-Rad, Hercules, CA, USA), with the threshold cycle number determined by use of the iCycler Software version 3.0. Reactions were performed in triplicate and the threshold cycle numbers were averaged. The results of the genes were normalized to housekeeping genes, *HPRT* for human and mouse, as described earlier [1]. Expression ratios were calculated according to the formula $2^{(Rt-Et)}/2^{(Rn-En)}$ [1], where *Rt* is the threshold cycle number for the reference gene observed in the test samples, *Et* is the threshold cycle number for the experimental gene observed in the test samples, *Rn* is the threshold cycle number for the reference gene observed in the reference samples, and *En* is the threshold cycle number for the experimental gene observed in the reference samples. *Rn* and *En* values were calculated as an average of all reference samples.

Rescue of secreted TFF1 protein in 3D organoid cultures

To confirm the role of TFF1 in regulating STAT3 in 3D organoid cultures, we performed a rescue experiment using TFF1 recombinant protein (Origene) or conditioned media from AGS-pcDNA and AGS-TFF1 cells as described before [2]. 3D organoids derived from the pyloric antral region of

TFF1-KO mice were plated in 24-well plates for 7 days to allow them to form. Two days before collecting the organoids, the culture media was supplemented with TFF1 recombinant protein (400ng.ml^{-1}) or replaced with either conditioned media from AGS-pcDNA or AGS-TFF1 cell line cultures. The organoids were collected, fixed in 10% formalin, embedded in paraffin, and blocks cut to 5 µm sections for immunofluorescence staining. For the *in vitro* experiment, AGS parental cells were treated with conditioned media from AGS-pcDNA or AGS-TFF1 cells for 24 h.

Enzyme-linked immunosorbent assay

The ELISA for human IL6/IL6Rα was performed following the manufacturer's instructions (R&D Systems, Minneapolis, MN). Briefly, 96-well microplates were coated with human IL6/IL6Rα capture antibody overnight at room temperature. After blocking, 100ul of the samples were added and incubated for 2 h. Next, biotinylated mouse IL6/IL6Rα was used as a detection antibody, followed by incubation with streptavidin-horseradish peroxidase (R&D Systems). Enzymatic reaction was performed using the peroxidase substrate BM and the absorbance was read at 450 nm on the FluoStar Optima plate reader (BMG LabThec).

TFF1 recombinant and TFF1 Neutralization antibody

AGS cells were cultured in Ham's F-12 supplemented with 10% FBS. After 48 h, cultured media was replaced with media containing recombinant TFF1 protein (400ng.ml^{-1}) and/or anti-TFF1 neutralizing antibody (600ng.ml^{-1}) and incubated overnight. Next day, cells were stimulated with IL6 (100ng.ml^{-1}) for 30 min. Cells were collected, lysed and subjected to Western blot analysis.

Transfection with GP130 plasmid

For adenovirus infection, AGS cells were infected with TFF1 or control adenoviruses (5MOI). The next day, cells were transiently transfected with 500ng of GP130 (IL6ST) expression plasmid (Addgene, Cambridge, MA) using FuGENE 6 according to the manufacturer's instructions (Roche Applied Science). After 24 h, cells were stimulated with IL6 (100ng.ml^{-1}) for 30 min. Cells were collected, lysed and subjected to Western blot analysis.

For recombinant TFF1 protein, AGS cells were cultured in Ham's F-12 supplemented with 10% FBS for 24 h. Next day, cells were transfected with 500ng of GP130 (IL6ST) expression plasmid. After 24 h, cultured media was replaced with media containing only recombinant TFF1 protein (400ng.ml^{-1}) and incubated overnight. The following day, cells were stimulated with IL6 (100ng.ml^{-1}) for 30 min. Cells were collected, lysed and subjected to Western blot analysis.

IL6/IL6Rα recombinant complex protein treatment

For adenovirus infection, AGS cells were infected with TFF1 or control adenoviruses (5MOI). After 48 h, cells were treated overnight with Human IL6/IL6Rα complex recombinant protein (10ng.ml^{-1}) (R&D Systems). Next day, cells were stimulated with IL6 (100ng.ml^{-1}) for 30 min. Cells were collected, lysed and subjected to Western blot analysis.

For recombinant TFF1 protein, AGS cells were cultured in Ham's F-12 supplemented with 10% FBS. After 48 h, cultured media was replaced with media containing recombinant TFF1 protein (400ng.ml^{-1}) and Human IL6/IL6Rα complex recombinant protein (10ng.ml^{-1}) and incubated overnight. Next day, cells were stimulated with IL6 (100ng.ml^{-1}) for 30 min. Cells were collected, lysed and subjected to Western blot analysis.

Gene Set Enrichment Analysis (GSEA)

Gene set STAT3_SIGNALING from MSigDB (v5.1) was analyzed with Gene Set Enrichment Analysis (GSEA) [3], using reported data sets [4]. Genes were ranked from high (left) to low (right) by log2 fold change, with STAT3-signaling genes indicated by vertical lines in the middle row. The ranking of the pathway was summarized by the enrichment score (ES) most distant from 0. A positive ES indicates that the pathway is enriched by up-regulated genes.

Supplementary references

1. El-Rifai W, *et al.* Gastric cancers overexpress S100A calcium-binding proteins. *Cancer Res* **62**, 6823-6826 (2002).

2. Soutto M, *et al.* Loss of TFF1 is associated with activation of NF-kappaB-mediated inflammation and gastric neoplasia in mice and humans. *J Clin Invest* **121**, 1753-1767 (2011).

3. Liberzon A, Birger C, Thorvaldsdottir H, Ghandi M, Mesirov JP, Tamayo P. The Molecular Signatures Database (MSigDB) hallmark gene set collection. *Cell Syst* **1**, 417-425 (2015).

4. Chen Z, *et al.* Integrated expression analysis identifies transcription networks in mouse and human gastric neoplasia. *Genes Chromosomes Cancer* **56**, 535-547 (2017).

Supplementary Figures

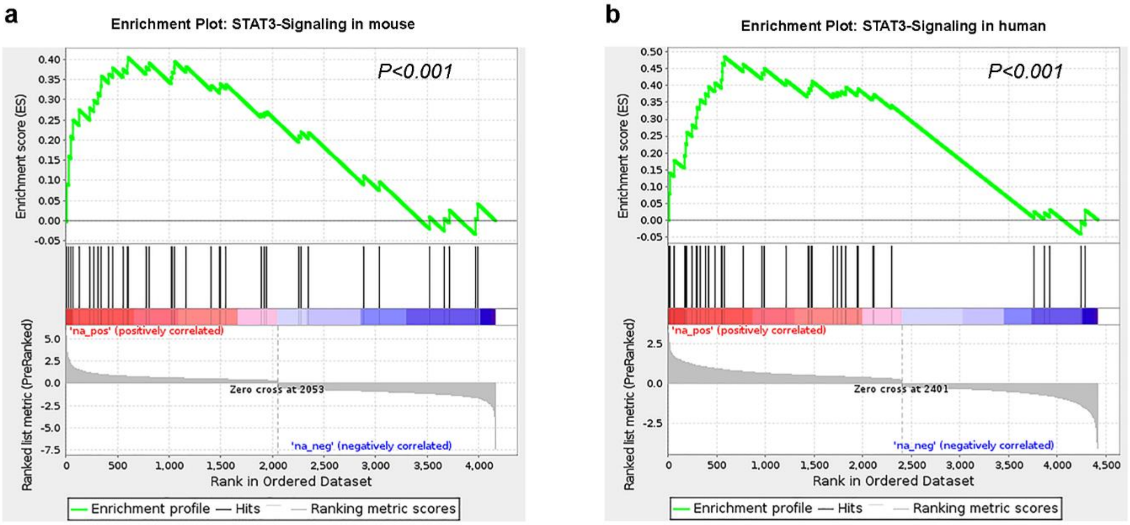

Supplemental Figure 1, Soutto et al.

Supplementary Figure 1. GSEA demonstrating strong association with STAT3 dependent gene set signatures in **(a)** TFF1-KO mouse gastric tissue**,** (*FDR=0.00749, P=0.00069)* **(b)** and human gastric cancer samples (*FDR=0.00174; P=0.00022*), *FDR*: False Discovery Rate.

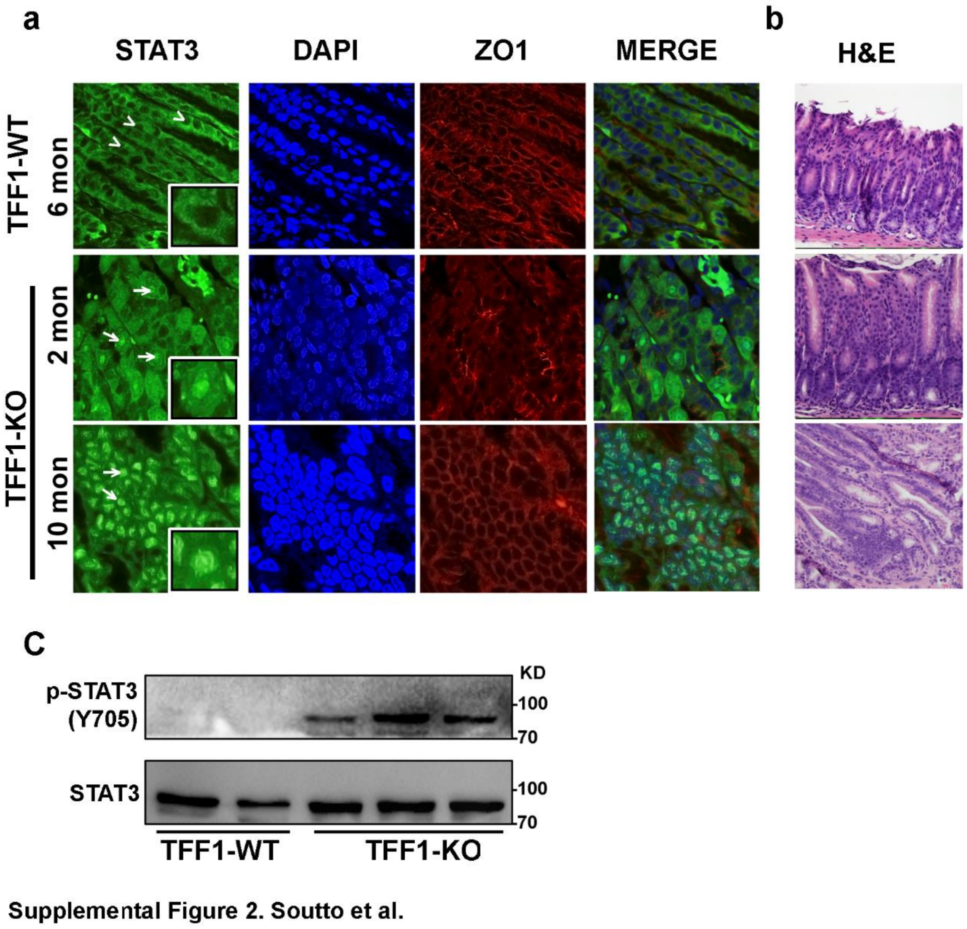

Supplementary Figure 2. TFF1 loss induces STAT3 nuclear localization in mouse gastric epithelial cells. (a) Immunofluorescence staining of p-STAT3 (Y705) in the antropyloric region of glandular stomach of the TFF1-KO mice showing nuclear staining in TFF1-KO *as early as* 2 months, as well as in 6 and 10 months age, but not in age-matched TFF1-WT mice. 4',6' Diamino-2-phenylindole (DAPI) (blue) was used as a nuclear counterstain and ZO1 (red) immunostaining was used as an epithelial cell marker, original magnification (x600) and insets (x1200). **(b)** H&E staining representing histological features of glandular gastric mucosa of corresponding ages, original magnification x200. **(c)** Western blot analysis showing protein levels of phospho-STAT3 (Y705) and total STAT3 in TFF1-WT and TFF1-KO gastric mouse tissue.

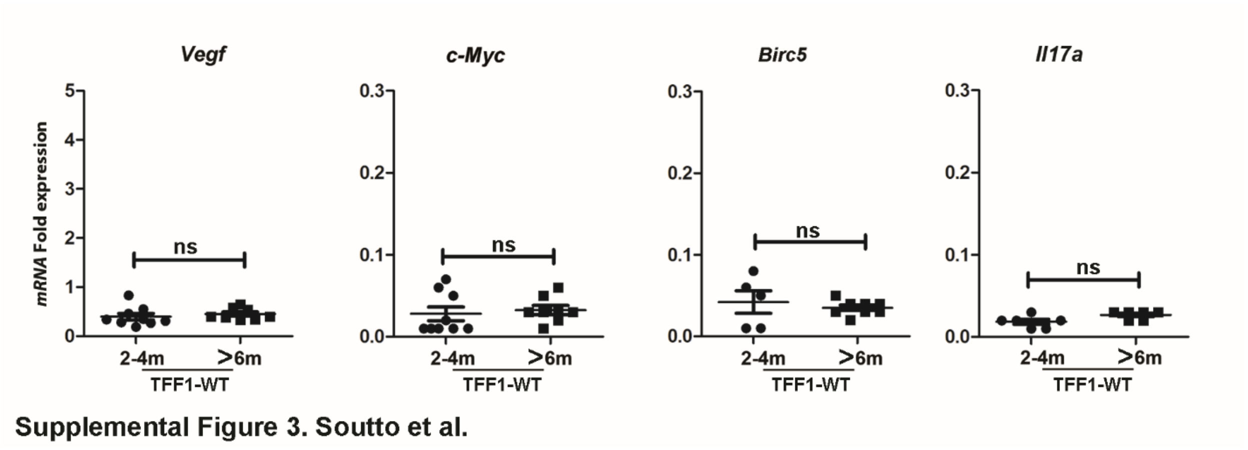

Supplementary Figure 3. The mRNA expression of STAT3 target genes in TFF1-WT gastric mouse tissue different ages. Quantitative real-time PCR analysis demonstrated no significant changes of mRNA expression of STAT3 target genes (*Vegf, c-Myc, Birc5 and Il17a*) in gastric tissues from TFF1-WT mice at different age 2-4 months and 6 month and up.

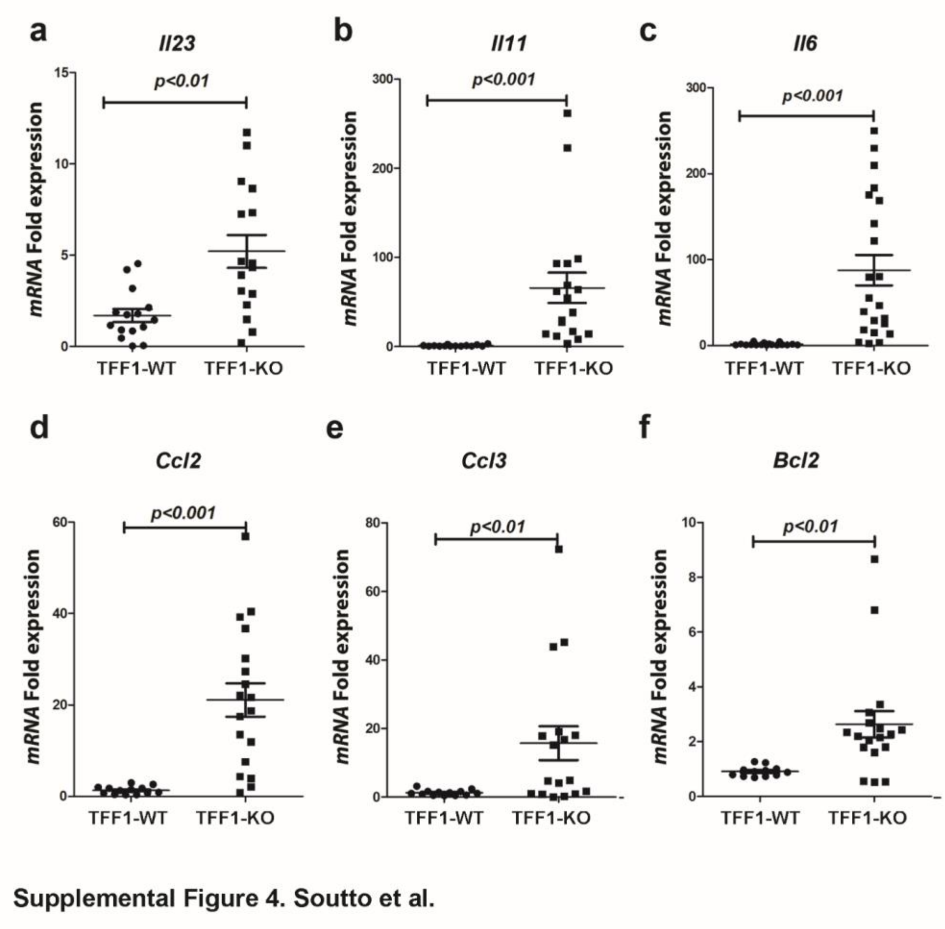

Supplementary Figure 4. Loss of TFF1 promotes increase of mRNA expression of STAT3 target genes in TFF1-KO gastric mouse tissue. (a-f) Quantitative real-time PCR analysis demonstrated upregulation of mRNA expression of STAT3 target genes (*Il23, Il11, Il6, Ccl2, Ccl3 and Bcl2*) in gastric tissues from the TFF1-KO mice (n=21) as compared with normal gastric tissues from TFF1-WT mice (n=15). **p<0.01 and ***p<0.001 by 2-tailed Student's t test.

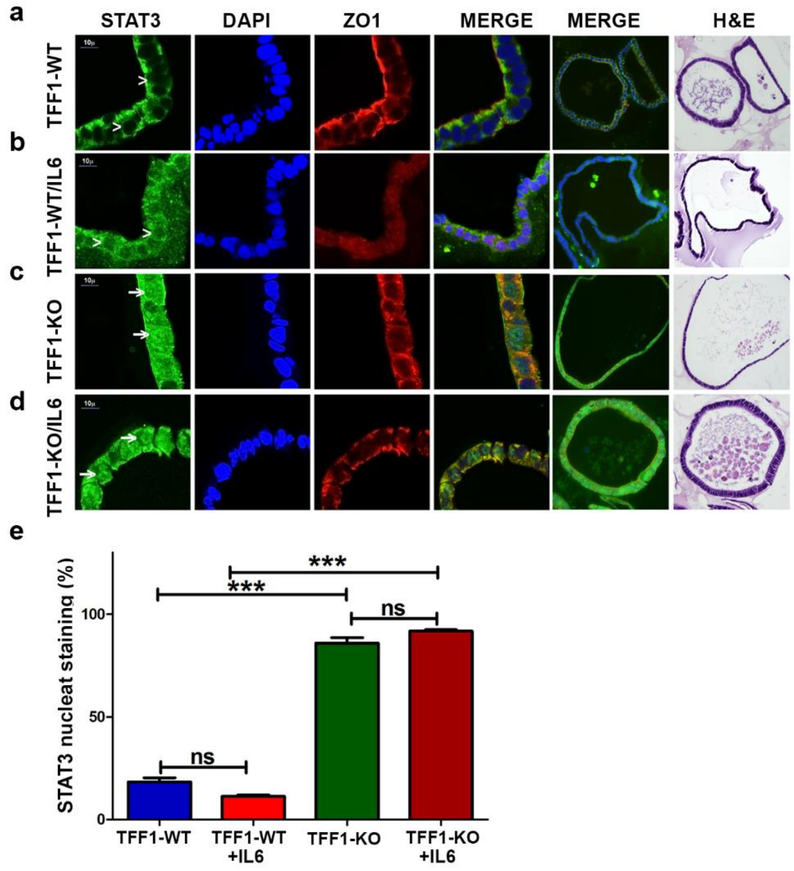

Supplemental Figure 5. Soutto et al.

Supplementary Figure 5. IL6-induced nuclear localization of STAT3 is suppressed in TFF1-WT but not TFF1-KO organoids. (a-d) Immunofluorescence staining of p-STAT3 gastric organoids derived from antropyloric glands of TFF1-WT (upper panels) and TFF1-KO (lower panels). **(a)** TFF1-WT gastric organoids showed no p-STAT3 nuclear staining (arrowheads). **(b)** Treatment with IL6 showed lack of nuclear p-STAT3, following IL6 treatment, similar to non-treated organoids (arrowheads). **(c-d)** In TFF1-KO mouse gastric organoid, the cells showed nuclear p-STAT3 similar to the IL6-treated samples (arrows). Nuclear localization of STAT3 is shown in green. 4',6' Diamino-2-phenylindole (DAPI) (blue) was used as a nuclear counterstain and ZO1 (red) immunostaining was used as an epithelial cell marker. H&E staining is presented on the right of each panel. Scale bars 10µm and original magnification for the entire organoid is shown at x600. **(e)** Graph showing the quantification of nuclear p-STAT3 positive cells in at least 4 counted organoid glands presented as percentage ± SEM (right upper panel) (at least 25-50 cells in each organoid). ****P<0.001* by 2-tailed Student's t test, ns: non-significant with *P>0.05*.

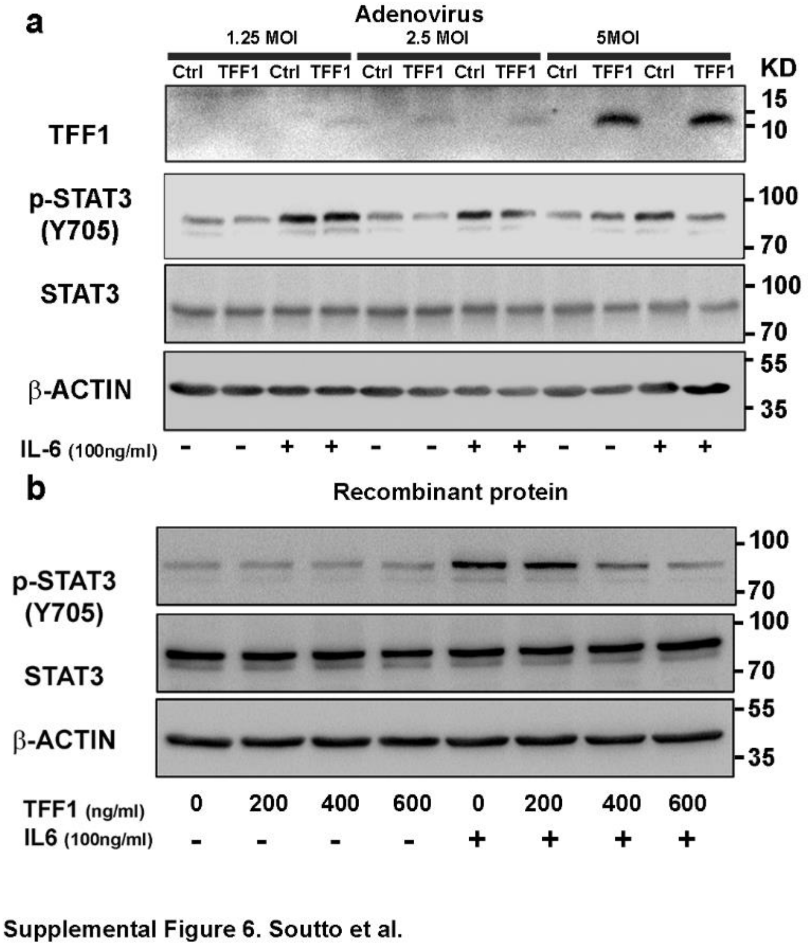

Supplementary Figure 6. TFF1 dose effect in suppressing IL6 mediated-STAT3 activation. (a) AGS cells were infected with different MOI (1.25, 2.5 and 5) of Control and TFF1 adenoviruses for 48h, and stimulated with IL6 (100ng/ml) for 30 min. 5 MOI of TFF1 adenovirus was the optimal dose to suppress IL6 mediated-STAT3 activation as compared to control adenovirus. **(b)** AGS cells treated over night with different doses of TFF1 recombinant protein (0, 200, 400 and 600ng/ml). next day, cells were stimulated with IL6 for 30 min. 400ng/ml was the optimal dose of recombinant TFF1 in decreasing IL6-mediated STAT3 phosphorylation. B-ACTIN was used as a control loading. The Western blot results represent 1 of 3 independent experiments.

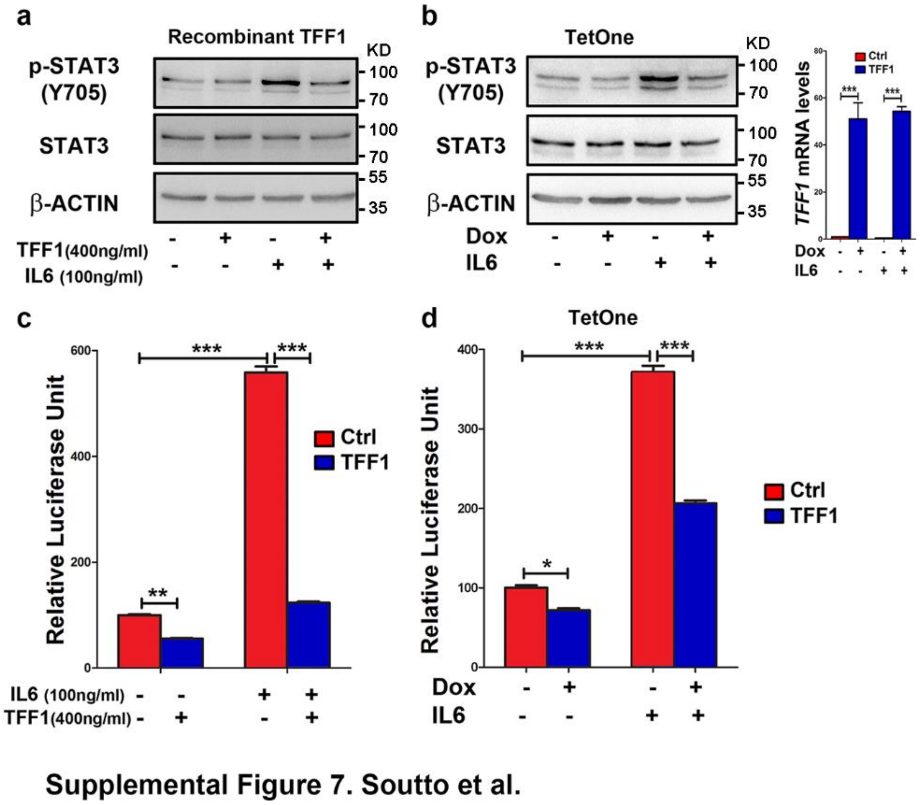

Supplementary Figure 7. Reconstitution of TFF1 decreases IL6–mediated phosphorylation and transcription activity of STAT3. (a-b) Western blots are shown following reconstitution of TFF1 and stimulation with IL6. **(a)** AGS cells treated with TFF1 recombinant protein 400ng/ml for 24h and stimulated with or without IL6 (100ng/ml) for 30 min. **(b)** AGS gastric cancer cells stably expressing TFF1 TetOne inducible system were stimulated with or without doxycycline (100ng/ml) for 48h and treated with IL6 (100ng/ml) for 30 min. The levels of TFF1 are shown by quantitative real time PCR analysis on the right side of each panel. The results are expressed as mean ± SEM of at least 3 independent experiments. **(c-d)** The luciferase reporter assay using STAT3–Luc reporter plasmid. **(c)** AGS cells were transfected with STAT3–luciferase reporter, next day cells were treated with TFF1 recombinant protein (400ng/ml) for 24h and stimulated with IL6 (100ng/ml) in the last 3h**. (d)** AGS cells stably expressing TFF1 TetOne inducible system were transfected with STAT3–luciferase reporter and stimulated with or without Doxycycline for 48h and treated or not with IL6 (100 ng/ml) for 3h. Data are presented as mean ± SEM of at least 3 experiments, with each condition performed in triplicate. *$P<0.05$ and ***$P<0.001$ by ANOVA Newman-Keuls Multiple Comparison Test.

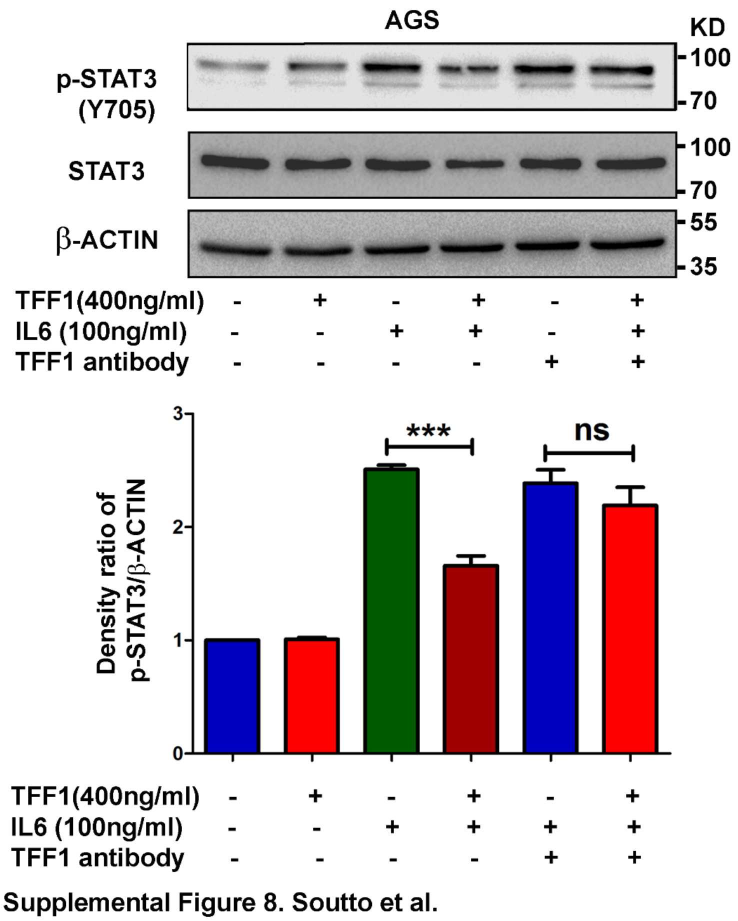

Supplementary Figure 8. TFF1 neutralizing antibody inhibits TFF1 mediated suppression of STAT3 activation. AGS cells were incubated for 24 hours with or without TFF1 recombinant protein (400ng/ml) combined with or without TFF1 neutralizing antibody (600ng/ml). Next day, cells were stimulated or not with IL6 (100ng/ml). TFF1 recombinant protein combined with neutralizing antibody showed no significant difference on STAT3 phosphorylation as compared to the corresponding control after IL6 stimulation. The results are expressed as mean ± SEM of at least 3 independent experiments.

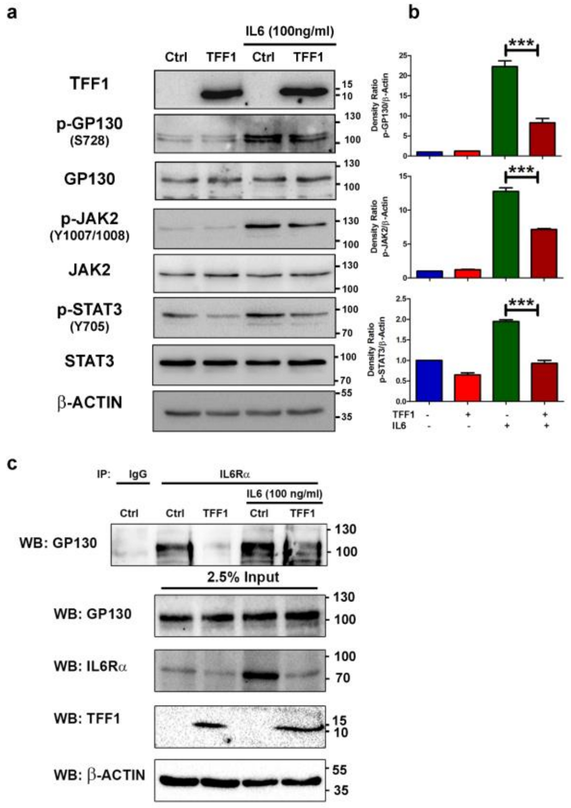

Supplemental Figure 9. Soutto et al.

Supplementary Figure 9. TFF1 negatively regulates IL6–induced STAT3 activation through GP130/IL6Rα axis in STKM2 gastric cancer cell lines. (a) Western blot analysis using STKM2 cell lines infected with control or TFF1 adenovirus. After stimulation with IL6 (100ng/ml) for 30 min, TFF1 expressing cells showed a significant decrease of p-STAT3 (Y705), p-GP130 (S782) and p-JAK2 (Y1007/Y1008) protein levels as compared to control cells. **(b)** The relative intensity ratio of p-JAK2/β-Actin, p-G130/β-Actin and p-STAT3/ β-Actin were calculated by Image-lab software from BioRad. The results are expressed as mean ± SEM of at least 3 independent experiments. ****P<0.001* by 2-tailed Student's t test **(c)** Immunoprecipitation and Western blot analysis following IL6Rα pulldown using STKM2 cells infected with TFF1 or control adenoviruses (5 MOI), with or without treatment with IL6 (100 ng/ml) for 30 minutes. The first lane exhibits STKM2 control following immunoprecipitation with mouse IgG control antibody. All immunoprecipitations and their corresponding input samples were subjected to immunoblotting with rabbit polyclonal antibody against GP130 and IL6Rα. The expression of TFF1 and equal amounts of protein loading were confirmed in the input samples.

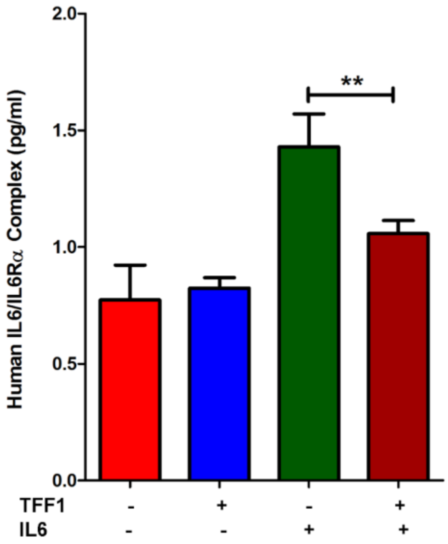

Supplemental Figure 10. soutto et al.

Supplementary Figure 10. TFF1 decreases formation of IL6/IL6Rα complex after IL6 stimulation. AGS cells were infected with TFF1 or control adenoviruses (5MOI), for 48 hour and stimulated with IL6 (100ng/ml) for 3h. Supernatant was analyzed for IL6/IL6Rα complex using ELISA kit (R&D Systems).

Supplementary Table1. Oligonucleotide sequence of mouse and human qRT-PCR primers

Gene ID	Mouse Forward Primers	Mouse Reverse Primers
Vegf	*GGAGAGCAGAAGTCCCATGA*	*TCGGGGTACTCCTGGAAGAT*
Il17a	*CAGGACGCGCAAACATGA*	*GCAACAGCATCAGAGACACAGAT*
c-Myc	*TCCTGTACCTCGTCCGATTC*	*TCTTCAGAGTCGCTGCTGGT*
Bric5	*GAATCCTGCGTTTGAGTCGT*	*CGATGCGGTAGTTCTTGAGG*
Il23	*GCACCTGCTTGACTCTGACA*	*ATCCTCTGGCTGGAGGAGTT*
Il11	*TGCTGACAAGGCTTCGAGTA*	*GAGCTGTAAACGGCGGAGTA*
Il6	*AAGGAGTTCACGGTGTTGCT*	*AAGGTCAAGCTCCTCCTTCC*
Ccl2	*CCAGCAAGATGATCCCAATG*	*TTTTAATGTATGTCTGGACCCATTC*
Ccl3	*AGATTCCACGCCAATTCATC*	*CCCAGGTCTCTTTGGAGTCA*
Bcl2	*GAGCGTCAACAGGGAGATGT*	*CATGCTGGGGCCATATAGTT*
Hprt	*TATGCCGAGGATTTGGAAAA*	*ACAGAGGGCCACAATGTGAT*
**Gene ID**	**Human Forward Primers**	**Human Reverse Primers**
VEGF	*CCTCCGAAACCATGAACTTT*	*ATGATTCTGCCCTCCTCCTT*
c-MYC	*GGTAGTGGAAAACCAGCAG*	*CAGCAGCTCGAATTTCTTC*
CXCL10	*GCTGATGCAGGTACAGCGT*	*CACCATGAATCAAACTGCGA*
IL17A	*CTGTGTCACCCCGATTGTC*	*TTGAAGGATGAGGGTTCCTG*
TFF1	*GGTCCTGGTGTCCATGCTG*	*ACAGCAGCCCTTATTTGCAC*
HPRT	*ACCCTTTCCAAATCCTCAGC*	*GTTATGGCGACCCGCAG*

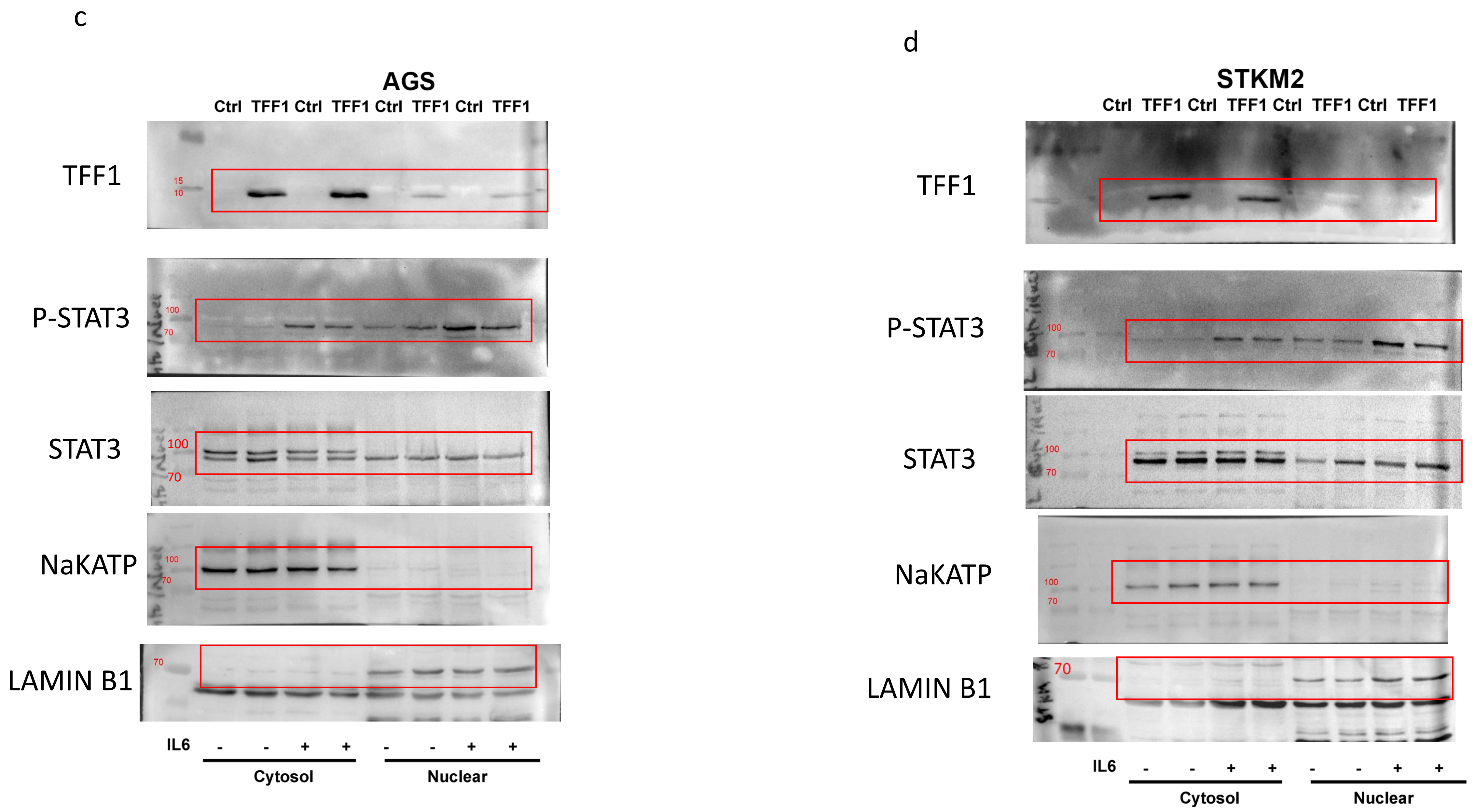

Figure 4. Soutto et al.

AGS	Control Adenovirus				TFF1 Adenovirus			
Untreated	39.76471	44.84211	43.03636		25.80556	27.28947	34.07692	
IL6	152.4032	155.9032	165.377	155.6833	99.14286	94.66667	84.07143	124.1935

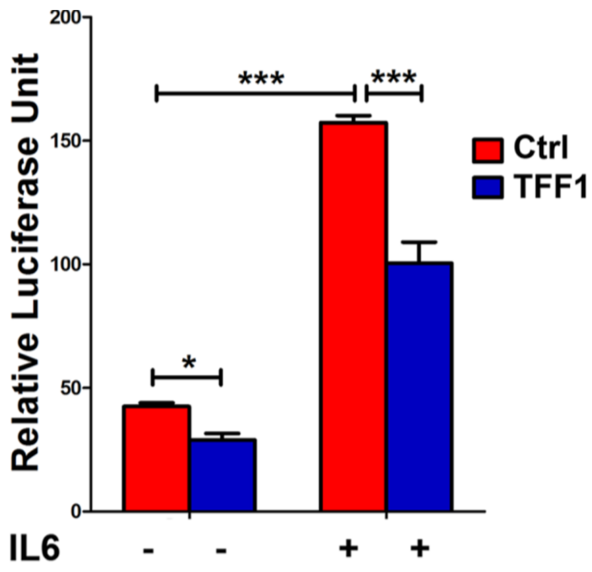

STKM2	Control Adenovirus				TFF1 Adenovirus			
Untreated	29.3913	25.2	32.06667	27.02174	16.04348	12.65455	15.53333	16.13043
IL6	56.62745	59.44	59.05769	60.06383	24.29412	25.92	22.53846	23.68085

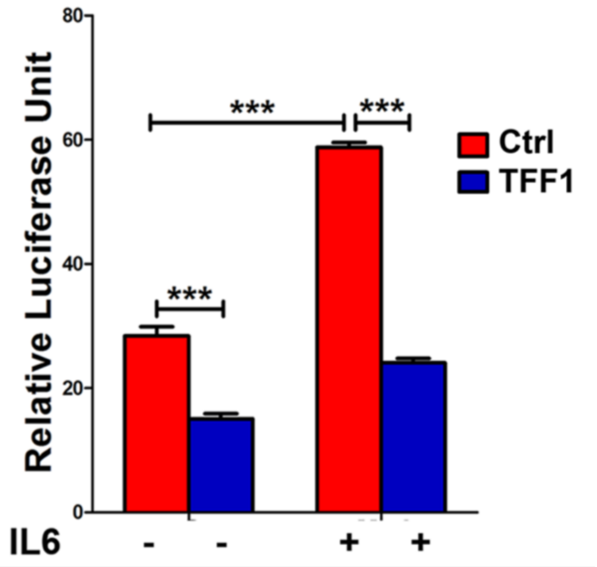

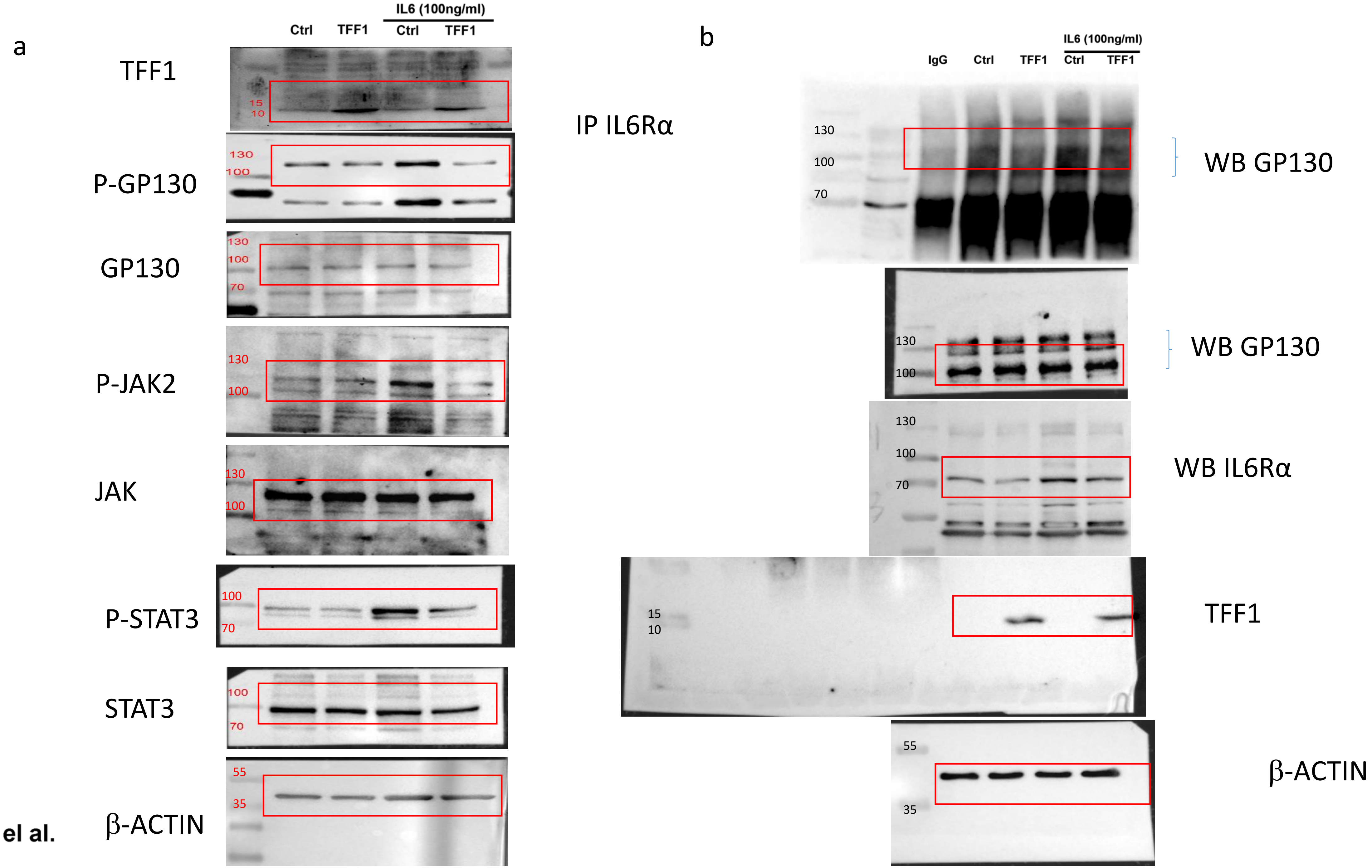

Figure 7. Soutto el al.

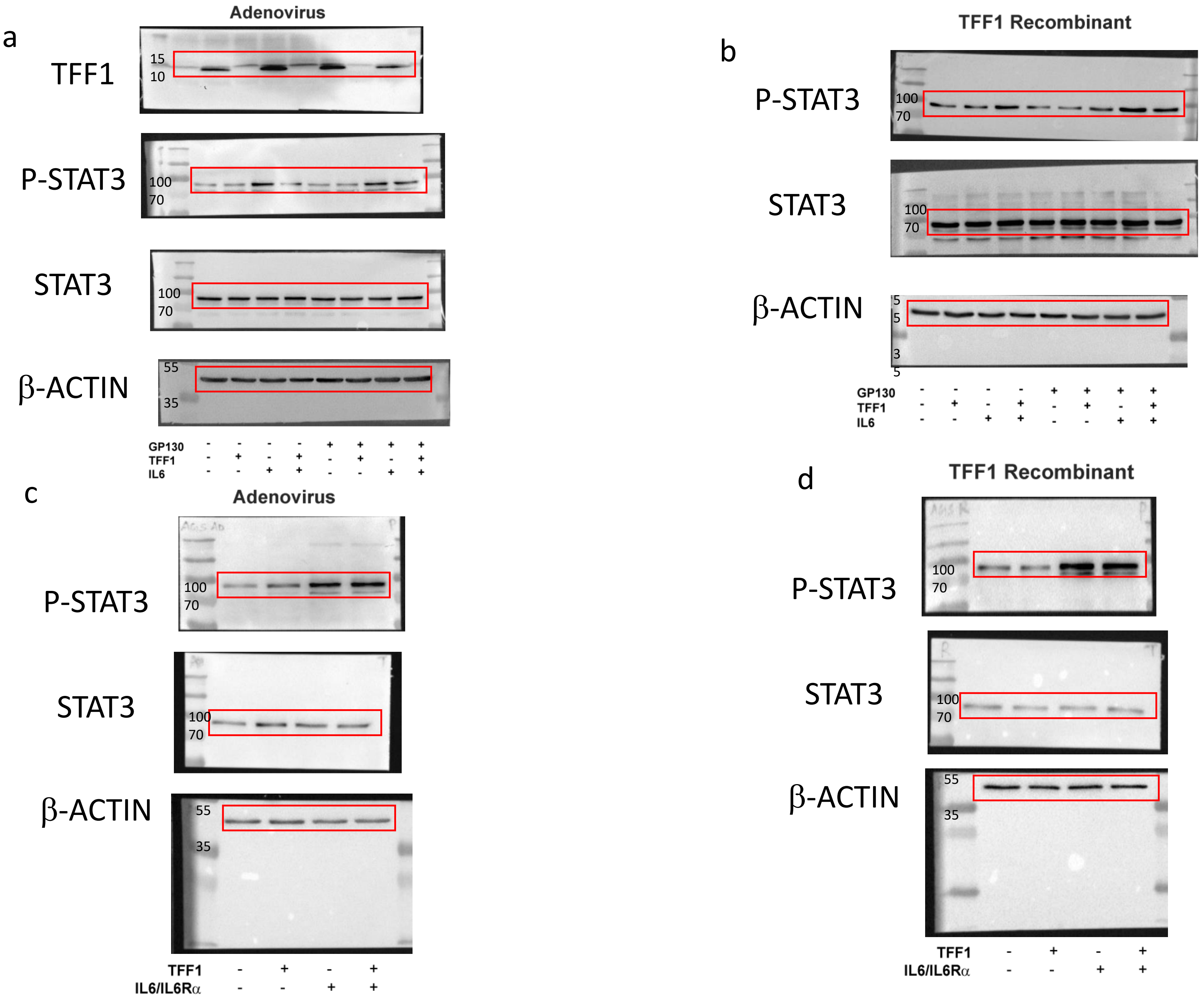

Figure 8. Soutto et al.

c

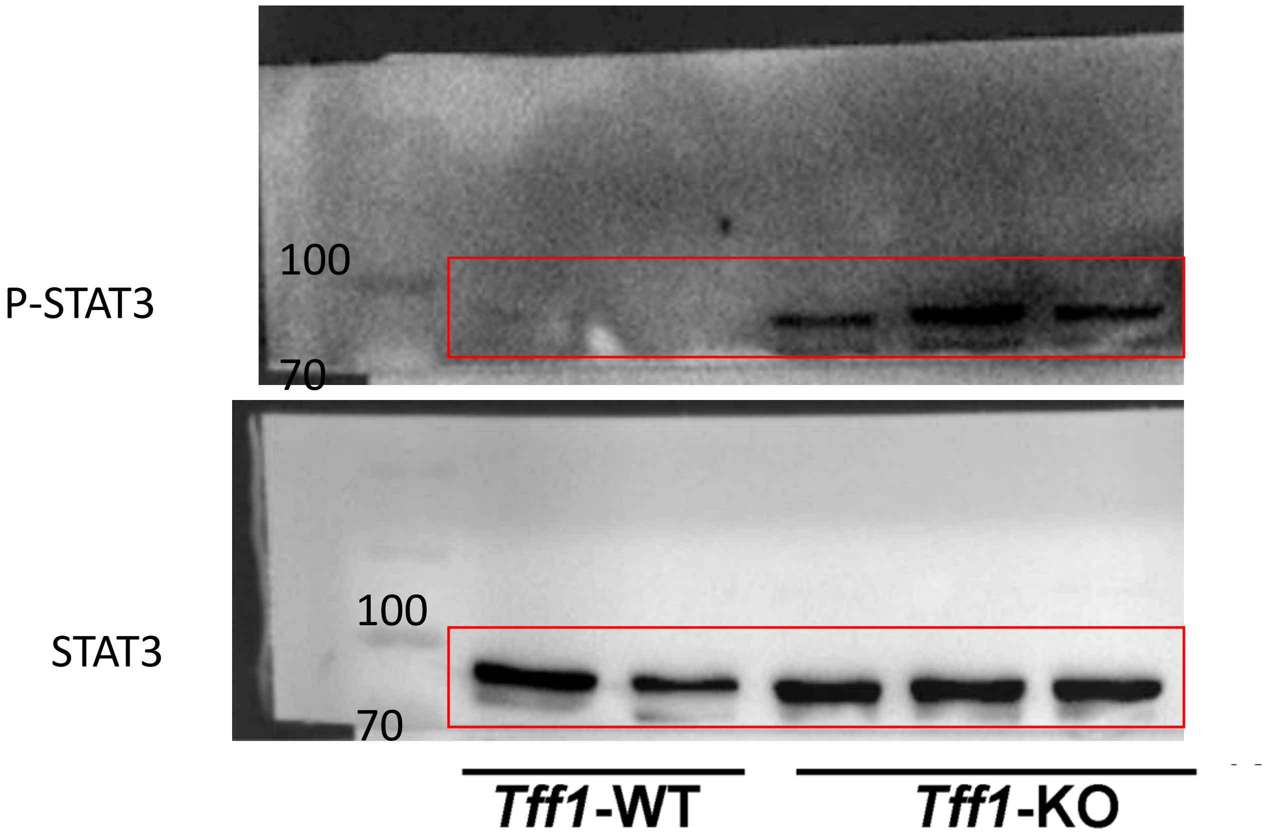

Supplemental Figure 2. Soutto et al.

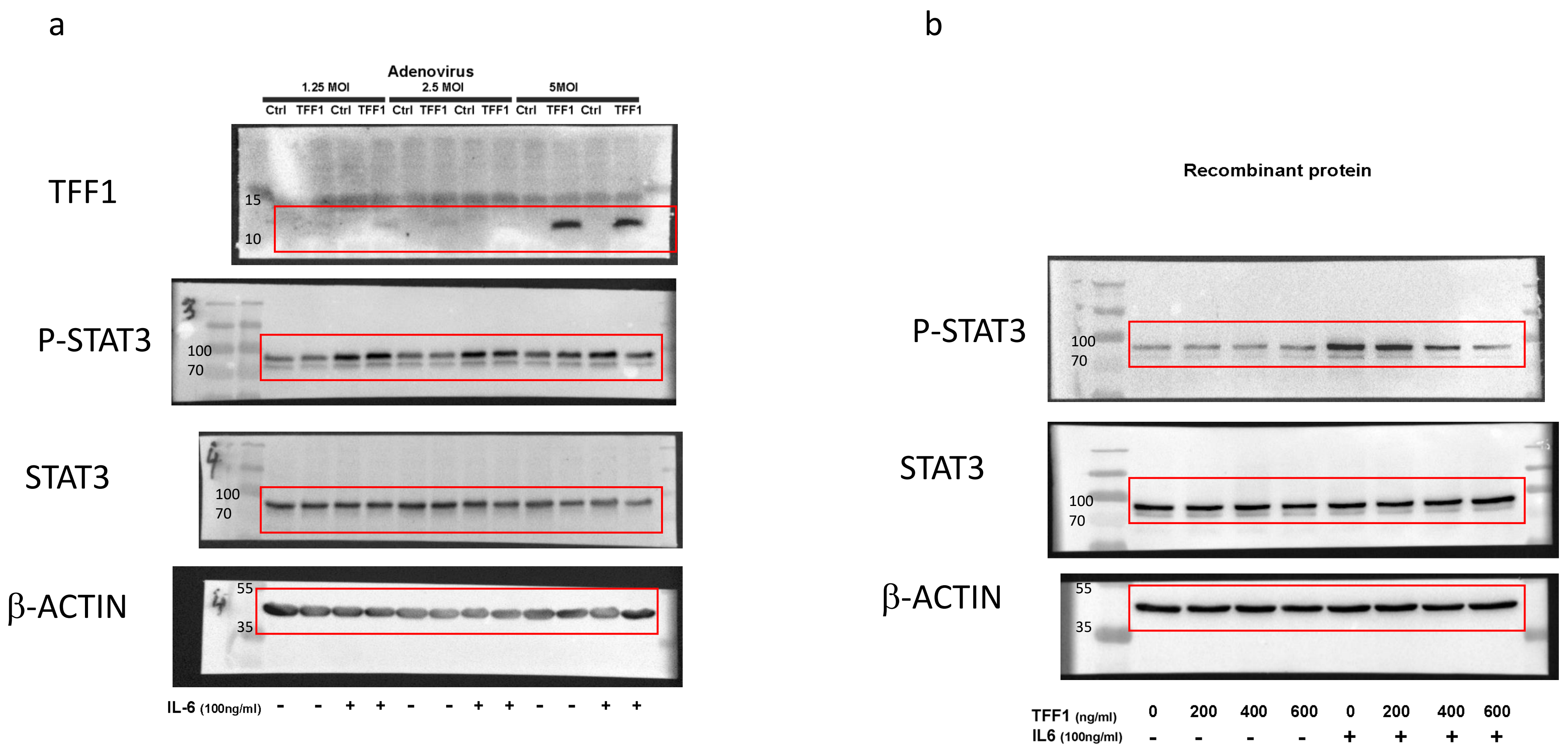

Supplemental Figure 6. Soutto et al.

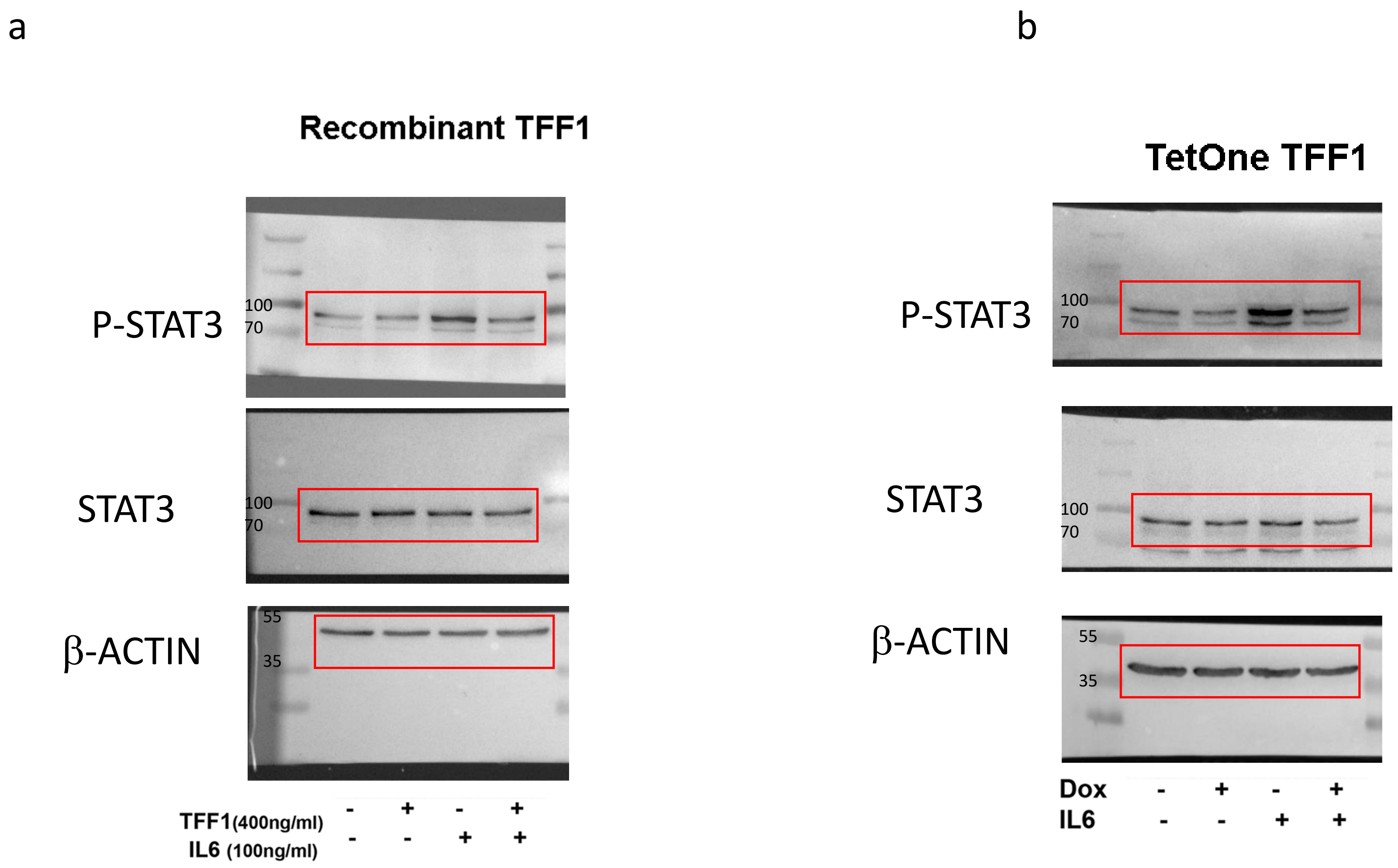

Supplemental Figure 7. Soutto et al.

c

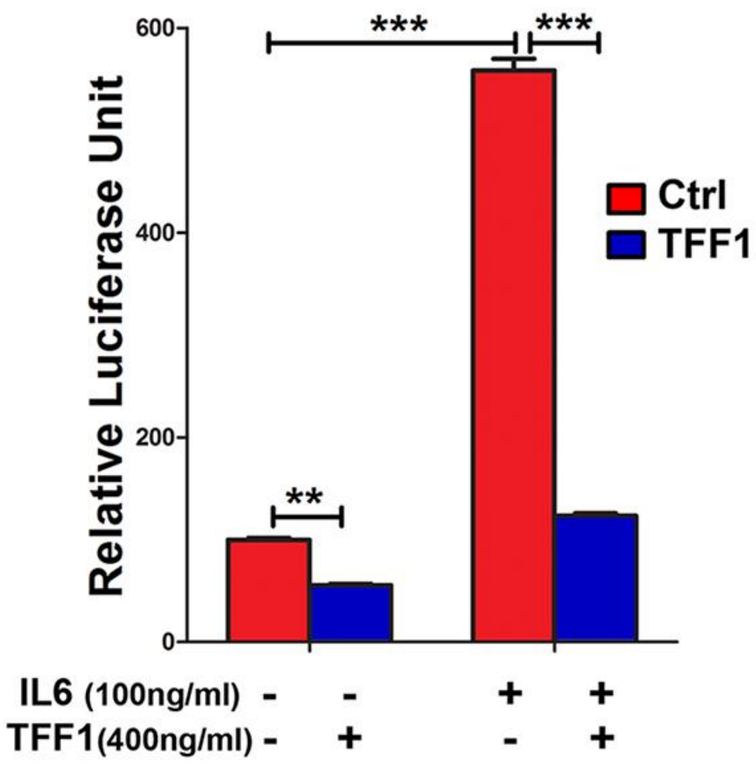

	Control Adenovirus			TFF1 Adenovirus		
Untreated	96.49624	101.9242	101.5795	56.86386	56.7777	52.64214
+IL6	559.5059	537.7942	577.5989	123.2912	119.4141	127.3406

d

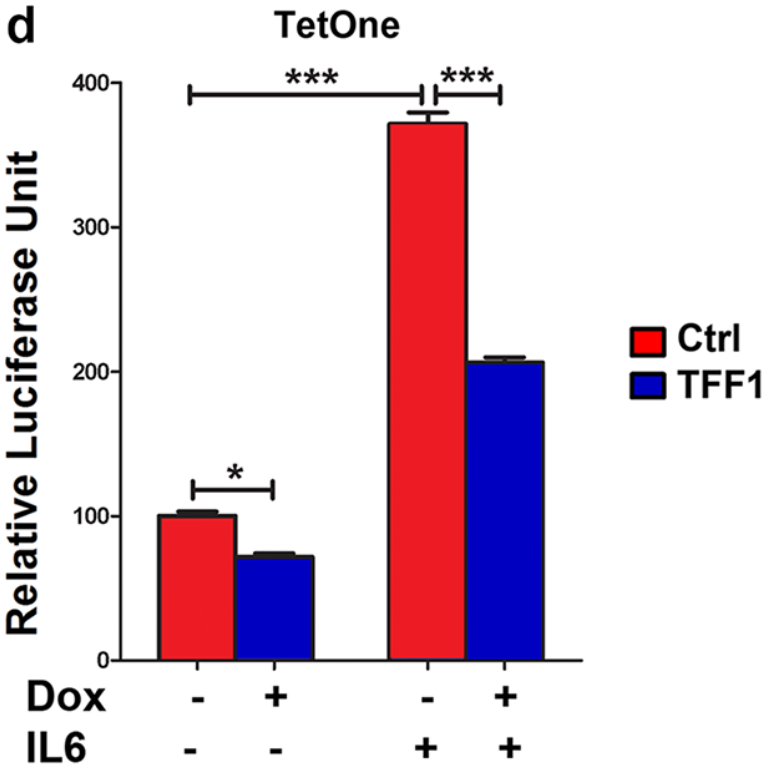

		IL6	
dox-	dox+	dox-	dox+
95.02064	66.79298	356.9969	200.6236
99.04791	73.68698	384.0848	212.7909
105.9316	74.58909	373.2757	205.6145

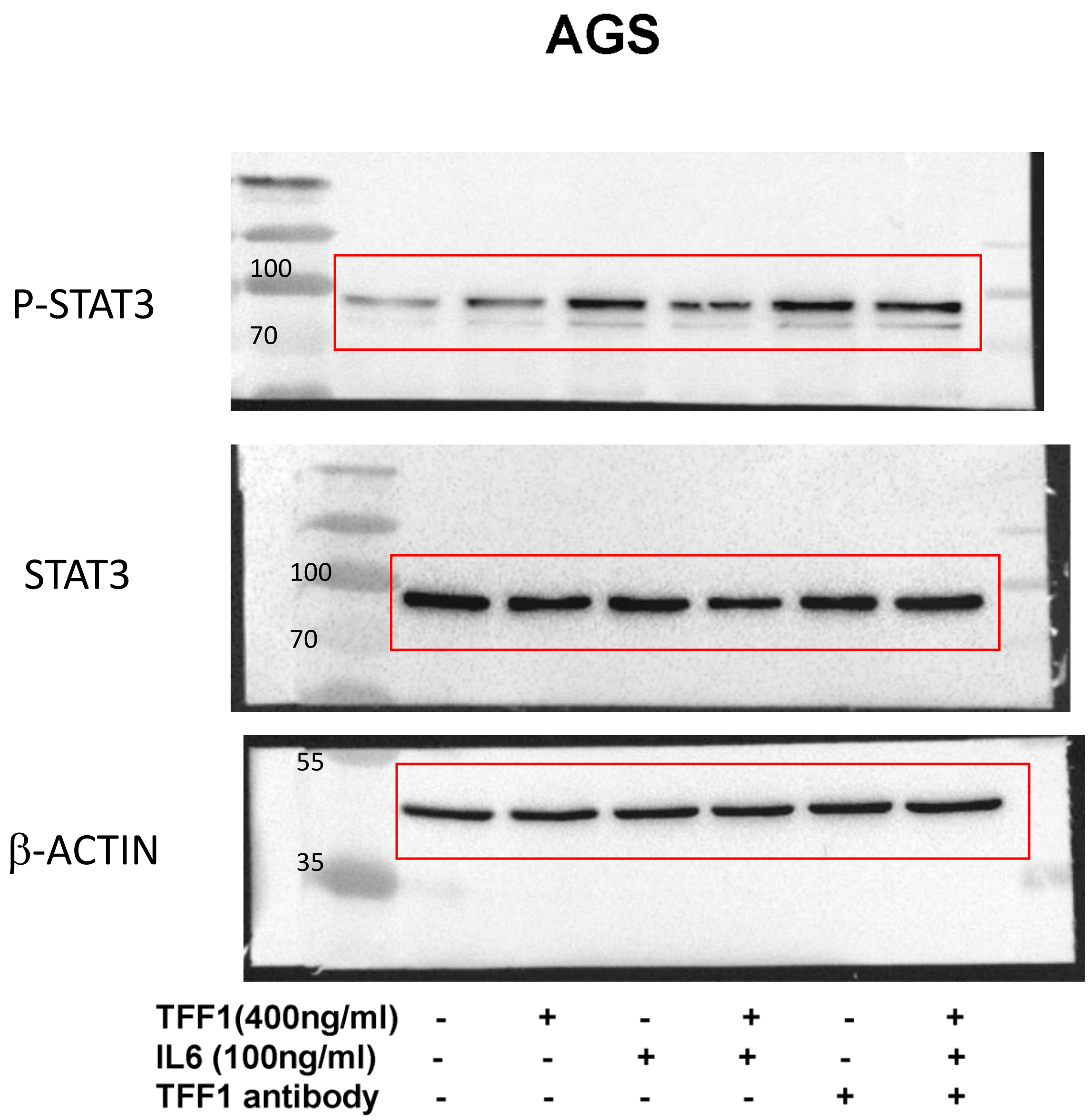

Supplemental Figure 8. Soutto et al.

Supplemental Figure 9. Soutto et al.

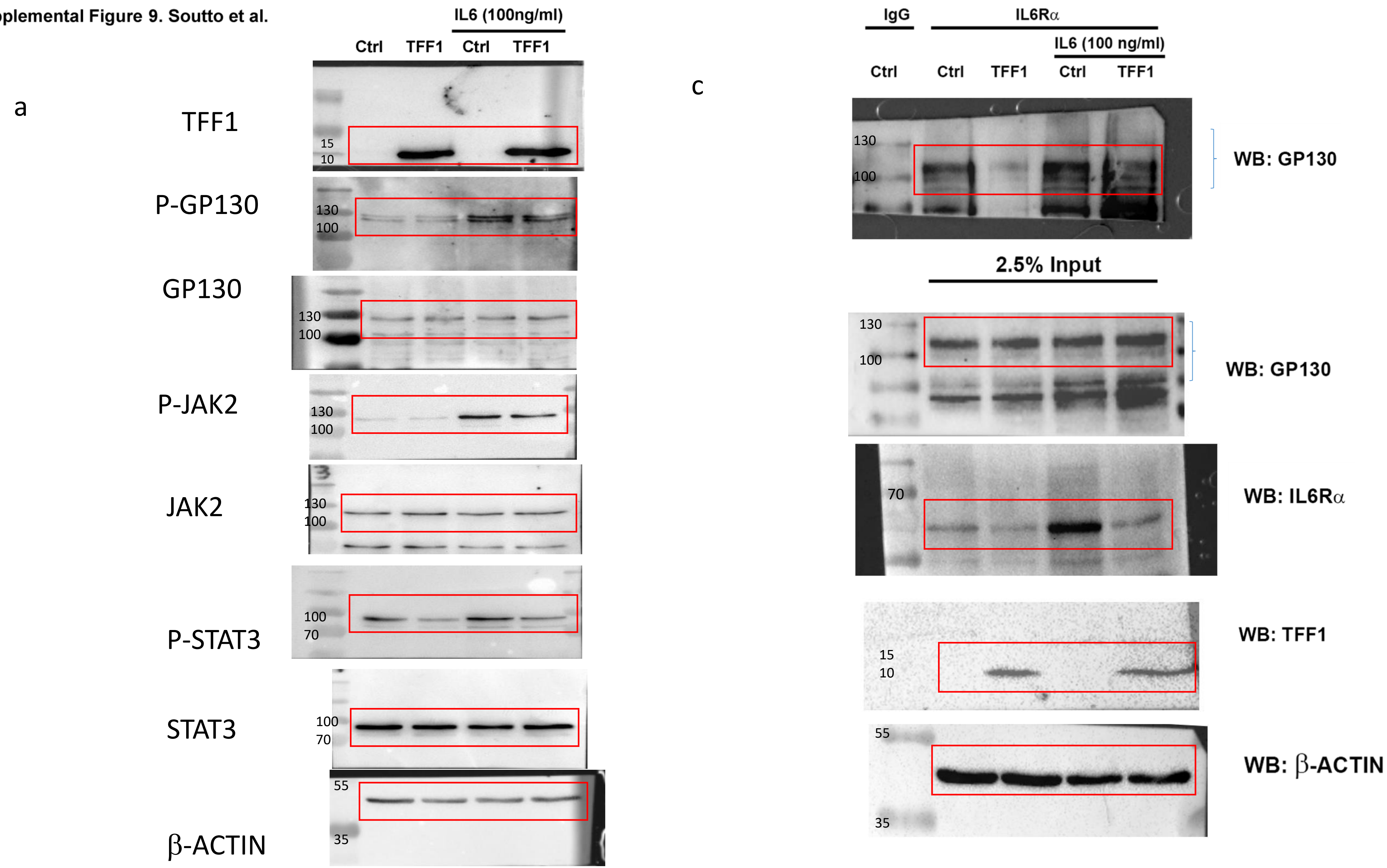

Supplement: Supplementary file 1 — Supplementary information [file 41467_2019_11011_MOESM1_ESM.pdf]
