# Supplementary material for: Activation of STAT3 signaling is mediated by TFF1 silencing in gastric neoplasia
Source: Nat Commun. 2019 Jul 10;10:3039. doi: 10.1038/s41467-019-11011-4 (PMC6620282; doi:10.1038/s41467-019-11011-4)
Supplement: Supplementary file 2 — Reporting Summary [file 41467_2019_11011_MOESM2_ESM.pdf]

nature research

Corresponding author(s): Dr. El Rifai

Last updated by author(s): 5/22/2019

# Reporting Summary

Nature Research wishes to improve the reproducibility of the work that we publish. This form provides structure for consistency and transparency in reporting. For further information on Nature Research policies, see Authors & Referees and the Editorial Policy Checklist.

## Statistics

For all statistical analyses, confirm that the following items are present in the figure legend, table legend, main text, or Methods section.

| n/a | Confirmed | |
|---|---|---|
| ☐ | ☒ | The exact sample size (*n*) for each experimental group/condition, given as a discrete number and unit of measurement |
| ☐ | ☒ | A statement on whether measurements were taken from distinct samples or whether the same sample was measured repeatedly |
| ☐ | ☒ | The statistical test(s) used AND whether they are one- or two-sided<br>*Only common tests should be described solely by name; describe more complex techniques in the Methods section.* |
| ☒ | ☐ | A description of all covariates tested |
| ☒ | ☐ | A description of any assumptions or corrections, such as tests of normality and adjustment for multiple comparisons |
| ☒ | ☐ | A full description of the statistical parameters including central tendency (e.g. means) or other basic estimates (e.g. regression coefficient) AND variation (e.g. standard deviation) or associated estimates of uncertainty (e.g. confidence intervals) |
| ☒ | ☐ | For null hypothesis testing, the test statistic (e.g. *F*, *t*, *r*) with confidence intervals, effect sizes, degrees of freedom and *P* value noted<br>*Give P values as exact values whenever suitable.* |
| ☒ | ☐ | For Bayesian analysis, information on the choice of priors and Markov chain Monte Carlo settings |
| ☒ | ☐ | For hierarchical and complex designs, identification of the appropriate level for tests and full reporting of outcomes |
| ☒ | ☐ | Estimates of effect sizes (e.g. Cohen's *d*, Pearson's *r*), indicating how they were calculated |

*Our web collection on statistics for biologists contains articles on many of the points above.*

## Software and code

Policy information about availability of computer code

| | |
|---|---|
| Data collection | GraphPad Prism software;<br>for 2D cultures we used ImageJ software;<br>Primer 3 (http://frodo.wi.mit.edu/cgi-bin/primer3/primer3_www.cgi<br>organoids were imaged on a Zeiss confocal microscope, using Zeiss ZEN software (Carl Zeiss Microscopy, Thornwood, NY).<br>For western blot analysis we used Image-lab software from BioRad |
| Data analysis | the analysis was used through the software mentioned above |

For manuscripts utilizing custom algorithms or software that are central to the research but not yet described in published literature, software must be made available to editors/reviewers. We strongly encourage code deposition in a community repository (e.g. GitHub). See the Nature Research guidelines for submitting code & software for further information.

## Data

Policy information about availability of data

All manuscripts must include a data availability statement. This statement should provide the following information, where applicable:

- Accession codes, unique identifiers, or web links for publicly available datasets
- A list of figures that have associated raw data
- A description of any restrictions on data availability

The data that support the findings of this study are available from the corresponding author upon reasonable request.

# Field-specific reporting

Please select the one below that is the best fit for your research. If you are not sure, read the appropriate sections before making your selection.

☒ Life sciences ☐ Behavioural & social sciences ☐ Ecological, evolutionary & environmental sciences

For a reference copy of the document with all sections, see nature.com/documents/nr-reporting-summary-flat.pdf

# Life sciences study design

All studies must disclose on these points even when the disclosure is negative.

| | |
|---|---|
| Sample size | For animals, we used more than 10 animals per group |
| Data exclusions | all the values were included |
| Replication | there was a triplicate for each experiment as described in the legends for each figure |
| Randomization | all samples collected were used for this study without any discrimination of sex |
| Blinding | All samples that were collected were analyzed. For the histology, pathologists were blinded to group of samples that were analyzed |

# Reporting for specific materials, systems and methods

We require information from authors about some types of materials, experimental systems and methods used in many studies. Here, indicate whether each material, system or method listed is relevant to your study. If you are not sure if a list item applies to your research, read the appropriate section before selecting a response.

## Materials & experimental systems

| n/a | Involved in the study | |
|---|---|---|
| ☐ | ☒ | Antibodies |
| ☐ | ☒ | Eukaryotic cell lines |
| ☒ | ☐ | Palaeontology |
| ☐ | ☒ | Animals and other organisms |
| ☒ | ☐ | Human research participants |
| ☐ | ☒ | Clinical data |

## Methods

| n/a | Involved in the study | |
|---|---|---|
| ☒ | ☐ | ChIP-seq |
| ☒ | ☐ | Flow cytometry |
| ☒ | ☐ | MRI-based neuroimaging |

## Antibodies

| | |
|---|---|
| Antibodies used | phospho-STAT3 (Y705) (cat# 9145S), phospho-JAK2 (Y1007/Y1008) (cat# 3771), STAT3 (cat# 12640), JAK2 (cat# 3230S) and β-actin (cat#3700) (purchased from Cell Signaling Technology, Beverly, MA). phospho-GP130 (Ser782) (cat#sc-377572) and GP130 (cat# sc-376280) were obtained from Santa Cruz Biotechnology, Inc. (Santa Cruz, CA). Anti-NaKATPase (cat# ab76020) (Abcam, Cambridge, MA) and lamin B (cat# sc-374015) (Santa Cruz, CA), anti IL6Rα (Cat#sc-373708,) TFF1 antibodies (cat#TA322883, Origene, Rockville, MD). |
| Validation | following manufacturer validation |

## Eukaryotic cell lines

Policy information about cell lines

| | |
|---|---|
| Cell line source(s) | AGS and STKM2 |
| Authentication | cells were authenticated usaing Cell Line Authentication Services, Genetica DNA Laboratories - a LabCorp brand Laboratory Corporation of America Holdings (LabCorp) |
| Mycoplasma contamination | cells were tested for mycoplasma negative |
| Commonly misidentified lines (See ICLAC register) | None |

## Animals and other organisms

Policy information about studies involving animals; ARRIVE guidelines recommended for reporting animal research

| | |
|---|---|
| Laboratory animals | study involved animals (mice) with different age as stated in methods |
| Wild animals | N/A |
| Field-collected samples | no samples were collected in Field |
| Ethics oversight | All animals were approved by the Institutional Animal Care and Use Committees at Vanderbilt University Medical Center and University of Miami |

Note that full information on the approval of the study protocol must also be provided in the manuscript.

## Clinical data

Policy information about clinical studies

All manuscripts should comply with the ICMJE guidelines for publication of clinical research and a completed CONSORT checklist must be included with all submissions.

| | |
|---|---|
| Clinical trial registration | N/A |
| Study protocol | N/A |
| Data collection | De-identified Human samples were anylyzed using tissue microarrays |
| Outcomes | N/A |
